# Quantifying the imputation paradox and XAI inconsistency in multi-source diabetes prediction: a 353,680-record leakage-free stacking ensemble with dynamic routing architecture

**DOI:** 10.3389/frai.2026.1898153

**Published:** 2026-08-07

**Authors:** M. Nanda Kishore, C. Navaneethan

**Affiliations:** School of Computer Science Engineering and Information Systems, Vellore Institute of Technology, Vellore, Tamil Nadu, India

**Keywords:** clinical decision support, decision curve analysis, diabetes prediction, dynamic routing pipeline, explainable AI, imputation paradox, leakage-free machine learning, LIME

## Abstract

Diabetes affects 537 million adults globally, a figure projected to reach 783 million by 2045. Despite over 4,200 ML prediction studies, clinical translation remains hindered by an over-reliance on benchmark datasets, unmeasured information costs of multi-source fusion, and untested XAI convergence assumptions. We address these issues by evaluating 353,680 records (from 455,446 candidates) across a Clinical-Biomarker Set (CBS) and a Lifestyle-Survey Set (LSS) using a strict leakage-free protocol. Under this protocol, normalisation, imputation, and SMOTE synthesis were fitted exclusively on training data. We formally define the Imputation Paradox as the AUC loss that occurs when critical diagnostic biomarkers are median-imputed under structural MNAR missingness. An Optuna-tuned stacking ensemble (Random Forest, XGBoost, LightGBM) achieves an AUC-ROC of 0.8481 on a held-out 70,736-sample test set. While a simple average baseline marginally exceeded stacking on pure discrimination (AUC 0.8521), stacking was retained for its superior precision (0.3439) and interpretable meta-learner trust weights, yielding a cross-validation AUC of 0.8491 ± 0.0014 and a Wilcoxon-significant improvement over baselines (*p* < 0.001, *n* = 1,000 replicates). A CBS sub-model trained on complete HbA1c and glucose data achieves an AUC of 0.9781. The resulting 0.130-unit gap quantifies this fusion-induced information cost. To recover this, our end-to-end dynamic routing pipeline routes 28.1% of patients to the CBS sub-model and 71.9% to the stacking ensemble, achieving a blended AUC of 0.8496. At a 0.30 mass screening threshold, the model identifies ~16,600 additional diabetic patients per million screened, with decision curve analysis confirming net benefit across the 5–30% threshold range. XAI conflict analysis yields Spearman correlations of *r* = −0.4561 (SHAP/permutation, *p* = 0.141), *r* = +0.2417 (SHAP/LIME, *p* = 0.737), and *r* = +0.1494 (permutation/LIME, *p* = 0.350). While non-significant at *n* = 12 features (critical *r* = ±0.576, *α* = 0.05), this provides preliminary directional evidence that these methods capture structurally different aspects of feature relevance.

## 1 Introduction

Regardless of how one frames the diabetes epidemic—be it a public health crisis, an economic catastrophe, or a preventable tragedy—the numbers are stark. The International Diabetes Federation’s 2021 Atlas (International Diabetes Federation, 2021) documents 537 million affected adults, a burden 74% larger than a decade earlier, and projects growth to 783 million by 2045. Healthcare expenditure has already surpassed USD 966 billion annually, and the 6.7 million deaths attributed to diabetes in 2021 exceeded the combined toll of HIV/AIDS, tuberculosis, and malaria (International Diabetes Federation, 2021). These outcomes are not inevitable.

The Diabetes Prevention Programme (Knowler et al., 2002) established that clearly: A trial spanning 3,234 participants across 27 centres found that lifestyle intervention cut progression from impaired glucose tolerance to clinical diabetes by 58%. The Diabetes Prevention Study (Tuomilehto et al., 2001) independently replicated that result. Before any intervention could be targeted, both trials first needed a reliable means of identifying who was at risk before the window for action closed. A review covering more than 4,200 published diabetes ML studies (Farooq and Khan, 2022) found a field that spent two decades refining benchmark performance rather than building tools with genuine clinical utility. Three structural failures explain the gap, and this study addresses all three.

### 1.1 The benchmark monoculture: a documented validity threat

Seventy-one percent of diabetes prediction studies published between 2018 and 2023 were trained on the Pima Indians Diabetes Dataset (Farooq and Khan, 2022)—768 records collected from Pima women in Arizona during the late 1980s (Smith et al., 1988). The problem is not that this dataset is small, though it is, but that models trained on it have consistently failed to transfer. Sun et al. (2021) measured the degradation: AUC drops of 0.06–0.11 when models built on historically and demographically specific cohorts face real-world validation populations. Weng et al. (2020) traced the mechanism: Distributional shifts in risk factor prevalence invalidate both learned feature weights and calibrated decision thresholds. Our decision to work with 353,680 contemporary records from two structurally distinct sources was directly motivated by this documented failure, not by any preference for large numbers.

### 1.2 Data leakage and the reproducibility crisis

Kapoor and Narayanan (2023) audited machine learning papers across medical domains and identified preprocessing-stage data leakage in a substantial proportion: Specifically, SMOTE synthesis or feature scaling applied before the train-test split. The consequence was AUC inflation of up to 0.05, sufficient to reverse model rankings. A worse model could appear better than a better one. Most diabetes ML publications carry this bias without disclosing it. Our pipeline was constructed as an explicit counter to this practice: Every imputation parameter, scaling factor, and synthetic sample was derived from training data only, with programmatic assertions enforced at each stage and documentation sufficient for independent reproduction (Kapoor and Narayanan, 2023).

### 1.3 The imputation paradox: a new concept

Here is a problem the field has not named, much less measured. When datasets with non-overlapping feature sets are merged, the absent columns are filled with median values. This routine practice quietly degrades the very signals most predictive of the outcome of interest. We term this the Imputation Paradox. In our study, HbA1c and blood glucose, the WHO’s definitional biomarkers for diabetes diagnosis (World Health Organization, 2019), are median-imputed across 71.7% of the fused training instances. A sub-model trained exclusively on complete, unimputed biomarker data achieves an AUC of 0.9781. The fused model achieves 0.8481. The 0.130-unit difference is the cost of fusion-induced imputation, quantified here for the first time in the diabetes prediction literature. Rubin’s MNAR framework (Rubin, 1976) explains why no imputation method can close this gap: The missingness mechanism is entirely determined by dataset membership, rendering the conditional distributions required for model-based imputation unidentifiable from observed data.

The broader phenomenon of a model with complete features outperforming a fused model where those features are structurally absent is a specific instance of the missing modality problem studied across multiple domains. The missing modality literature in medical imaging and multimodal learning has documented performance degradation under absent input channels. Jerez et al. (2010) showed that imputation strategy selection is consequential in clinical fusion once missingness exceeds 40%. Rubin’s MNAR framework (Rubin, 1976) establishes theoretically that structural missingness makes imputation unidentifiable. What the literature has not done, to our knowledge, is (1) formally define the case where fusion-induced imputation of diagnostically critical features produces lower performance than training on the smaller but complete source dataset; (2) measure this cost empirically as an AUC gap in a diabetes-specific tabular fusion context; or (3) show that the same MNAR mechanism that suppresses discriminative performance also suppresses SHAP attributions for the affected features, extending the cost from prediction accuracy into explainability. Our contribution is the definition, measurement, and naming of this specific pattern, not the claim that information loss under missing modalities was previously unknown.

### 1.4 XAI inconsistency: testing an unchallenged assumption

SHAP (Lundberg and Lee, 2017) and LIME (Ribeiro et al., 2016) appear routinely in clinical AI papers as evidence of model interpretability. Neither paper tests whether the two methods agree. That assumption should not be left unchallenged. SHAP computes exact Shapley values (Shapley, 1953), the weighted average marginal contribution of each feature across all possible feature coalitions. LIME constructs a local linear surrogate by perturbing instances near a query point (Ribeiro et al., 2016). Permutation importance shuffles individual features across the test set and measures the resulting accuracy decline (Breiman, 2001). These are three fundamentally different measurement operations. There is no theoretical ground for assuming they converge. Our Spearman inter-method agreement analysis yields a maximum correlation of *r* = 0.1086—not slight disagreement, but the first quantitative evidence that these methods are measuring different aspects of feature relevance in this domain, and that reporting only one of them gives clinicians a partial and potentially misleading picture.

### 1.5 Population-scale clinical motivation

The clinical consequences of the identification gap are concrete. At a mass screening threshold of 0.30, our model identifies an additional 1,174 diabetic patients per 70,736 screened, relative to the default operating point. Scaled to one million screened, this amounts to approximately 16,600 additional patients identified per screening cycle. Williams et al. (2004) documented the cost of undetected years: Diabetic retinopathy, the leading cause of new-onset blindness in working-age adults, progresses silently during the asymptomatic period, with irreversible structural changes present well before visual symptoms appear. NHS England data (NHS England, 2023) frames the economics: The burden of missed diagnoses through avoidable complications substantially exceeds the cost of a confirmatory HbA1c test.

### 1.6 Contributions of this paper

Five original contributions are reported: (1) A leakage-free multi-source fusion pipeline designed for reproducibility from the outset, not as an afterthought (Kapoor and Narayanan, 2023); (2) an Optuna-tuned stacking ensemble of Random Forest, XGBoost, and LightGBM that achieves an AUC of 0.8481 with the highest precision of all evaluated configurations (0.3439); ablation analysis shows RF + XGB pairwise averaging marginally exceeds it by AUC (0.853), while the stacking ensemble’s precision and interpretability properties justify its selection as the deployment architecture; (3) the first empirical quantification of the Imputation Paradox, with 0.130 AUC units attributable to MNAR-driven median imputation of diagnostic biomarkers; (4) the first Spearman correlation-based inter-method XAI agreement analysis in the diabetes prediction domain; and (5) a formally specified and empirically evaluated dynamic routing pipeline (Algorithm 1, Section 6.12) that routes 28.3% of patients to a CBS sub-model (AUC 0.9781) and 71.7% to the stacking ensemble (AUC 0.8481), achieving a blended end-to-end AUC of 0.8496 on the test set—a 0.0015 improvement over stacking-only deployment—with disagreement flags firing for 71.7% of patients served by the routing system. This Optuna-tuned stacking ensemble, whose Logistic Regression meta-learner provides human-readable trust weight coefficients (Table 1), offers transparency that satisfies clinical governance requirements under the EU AI Act by documenting exactly how the model architecture weighs expert opinions from diverse base learners.

**Table 1:** Fitted logistic regression meta-learner coefficients from stacking ensemble training.

Base learner	Coefficient *β*	Exp (*β*)	Relative weight
LightGBM	3.464292	31.9538	42.14%
XGBoost	3.702331	40.5417	45.03%
Random Forest	−1.054840	0.3482	12.83%
Intercept *β*	−3.036333	0.0480	

Input features are the out-of-fold probability predictions from each base learner on the SMOTE-balanced training partition (5-fold stratified CV, n_train = [verified value]). Coefficients are interpretable as trust weights: A larger positive *β* indicates that the meta-layer assigns greater weight to that learner’s probability estimates when forming the stacked prediction. Exp(β) represents the multiplicative change in predicted odds per unit increase in the corresponding base learner’s probability output. Relative weight = |β| / *Σ*|β| × 100.

## 2 Theoretical framework

### 2.1 Shapley values, SHAP, and interaction effects

The theoretical foundation of SHAP (Lundberg and Lee, 2017) stems from Shapley’s (1953) work in cooperative game theory. The analogy is useful: Each feature is treated as a player in a coalition, and the model’s output as the payoff to be fairly distributed among those players. A feature’s Shapley value is its average marginal contribution when features are added to the model in every possible ordering, a measure that accounts for how a feature performs both alone and in combination with every possible subset of other features. What makes this theoretically attractive, rather than merely intuitive, is the axiomatic foundation established by Lundberg and Lee (2017). SHAP is the unique additive attribution method satisfying local accuracy (the attributions sum to the model output), missingness (absent features receive zero attribution), consistency (a feature that contributes more in one model than another receives a higher attribution), and efficiency (attributions sum to the difference between the model output and its expected value). Heuristic importance scores satisfy none of these simultaneously. The TreeExplainer variant (Lundberg et al., 2020) makes exact Shapley computation tractable even for large ensembles by exploiting conditional independence within decision tree nodes. The extension to SHAP Interaction Values (Lundberg et al., 2020) matters considerably for interpreting our results. Rather than reporting a single attribution per feature, interaction values decompose each feature’s contribution into a standalone main effect and pairwise interaction terms with every other feature in the model. This decomposition explains the HighBP finding in Section 6.9, where SHAP assigns rank 1 and permutation importance assigns rank 12. HighBP does not rank first due to any strong independent effect; it ranks first due to its coalition with HighChol and hypertension. When those three features co-occur in a high-risk patient profile, HighBP’s conditional marginal contribution is substantially elevated relative to its unconditional effect. Permutation importance, which shuffles one feature at a time and cannot account for what correlated features substitute, misses this entirely. Both methods are answering valid questions; they are not answering the same one.

### 2.2 2LIME: model-agnostic local approximation and its theoretical limits

Ribeiro et al. (2016) developed LIME to solve a genuinely difficult problem: How do you explain the prediction of any black-box model when you have no access to its internals? The solution is local approximation. For a query instance x₀, LIME generates a set of perturbed neighbours, queries the model on each, and fits a locally weighted linear regression to approximate the model’s decision surface in the vicinity of x₀. The regression coefficients from that surrogate become the feature attributions for that instance. The approach has a real strength: It needs only query access to the model, making it applicable to any classifier regardless of architecture. However, that generality carries a cost, and in our setting, the cost is consequential. The local linear approximation is reliable only when the model’s decision surface near x₀ is itself approximately linear. For stacking ensembles operating in correlated clinical feature spaces, where non-linear interaction effects drive the bulk of model performance, LIME’s linear lens systematically underweights features whose importance is interaction-dependent rather than marginal. This is not a flaw in LIME’s design; it is an inherent property of local linear approximation in non-linear spaces. We treat this limitation not as a criticism of the method but as the mechanistic explanation for the disagreement we observe between SHAP and LIME in Section 6.9. A feature like HighBP, whose Shapley value is dominated by interaction terms with HighChol and hypertension, will receive a low LIME attribution because the local linear surrogate cannot capture coalition effects; it observes only the direct contribution near the query point. SHAP, by construction, integrates across all possible coalitions. Neither attribution is wrong. They are measuring different things, and that difference matters when the goal is clinical decision support rather than benchmark performance.

### 2.3 Permutation importance: global accuracy dependence

Permutation importance (Breiman, 2001) asks a population-level question: How much does overall accuracy depend on each feature? Feature j is shuffled across all test instances, the accuracy drop is measured, and this is averaged over repetitions. The result tells you how much the model globally relies on j, but here is the key limitation: When feature A is highly correlated with feature B, shuffling A allows the model to recover A’s information through B. Permutation importance for A drops, even if A has high SHAP values in interaction-rich profiles. This correlation–substitution mechanism, not model error, is why HighBP appears unimportant globally (permutation rank 12) while appearing locally critical (SHAP rank 1). Both results are valid. Understanding why they diverge is the contribution.

### 2.4 The MNAR structure and why imputation cannot recover it

Rubin (1976) classified missing data into three types: missing completely at random (MCAR), missing at random (MAR), and missing not at random (MNAR). Multiple imputation by chained equations (MICE) (van Buuren and Groothuis-Oudshoorn, 2011) and *k*-nearest-neighbour imputation (Zhang, 2016) are valid under MCAR and MAR because the missingness mechanism is, by definition, independent of the missing values. Our missingness is MNAR in a structural sense, which makes model-based imputation futile: HbA1c and blood glucose are absent from all 253,680 Lifestyle-Survey records, not because of any probabilistic data process, but because a telephone lifestyle survey never collected biomarkers. The missingness is categorical and entirely a function of the dataset a row originated from. The conditional distributions required for MICE or KNN to work are not identifiable from the observed data. Pilot tests confirmed what theory predicts: MICE improved fused AUC by less than 0.002 over median imputation. We chose median imputation because its operation is transparent.

### 2.5 Model calibration: Brier score and reliability diagrams

Discriminative performance and calibration are not the same thing, and in clinical settings, they are not interchangeable. AUC tells you whether the model ranks diabetic patients above non-diabetic ones, a useful property for ranking and prioritisation. Calibration tells you whether the model’s probability outputs are numerically trustworthy: When the model assigns a 0.70 probability of diabetes to a patient, does 70% of that group actually have the condition? The two can diverge substantially. A model achieving AUC 0.85 can simultaneously assign a 0.70 probability to patients whose actual event rate is 0.30—a miscalibration that becomes clinically dangerous when physicians use the probability estimate itself to decide whether to pursue confirmatory testing. Two complementary tools are used to assess calibration here. Reliability diagrams plot the mean predicted probability against observed event rates within equal-frequency bins (Platt, 1999), giving a visual account of where and how the model overestimates or underestimates risk. The Brier score (Brier, 1950), the mean squared difference between predicted probabilities and binary outcomes, provides a single scalar summary of overall calibration quality. Together, these give clinical implementers what AUC alone cannot: A picture of whether the model’s numerical outputs can be trusted at the individual patient level, not just at the population ranking level. Both metrics are reported in Section 6.10.

### 2.6 Net benefit analysis for clinical threshold justification

Precision-recall analysis is useful but does not fully address the question a clinician actually faces at the point of care. Knowing that a model achieves a certain recall at a given threshold does not indicate whether using that threshold produces better outcomes than simpler alternatives, such as recommending further testing for everyone or for no one. Vickers and Elkin’s decision curve analysis (DCA) (Vickers and Elkin, 2006) is designed to answer this more practically grounded question. DCA works by plotting net benefit across the full range of decision thresholds. Net benefit is defined as the number of true positives per patient screened, weighted by the relative cost of a false positive versus a false negative—a ratio that the clinician or decision-maker specifies based on the clinical context. By plotting this quantity for the model alongside the ‘treat-all’ and ‘treat-none’ baselines, DCA reveals at which thresholds the model actually adds value relative to the simplest conceivable alternatives. For diabetes screening, the clinically relevant threshold range is roughly 5–30% predicted probability. This is the zone where a general practitioner genuinely cannot make a confident recommendation about confirmatory HbA1c testing from clinical examination alone, and where a decision support tool could alter the outcome. A model that adds net benefit throughout this range, relative to both default strategies, provides evidence of clinical utility that AUC and F1 are structurally unable to supply. The decision curves for our model appear in Section 6.11.

## 3 Related work

### 3.1 Machine learning for diabetes risk: the Generalisability gap

The computational study of diabetes prediction often dates back to 1988, when Smith et al. (1988) applied the ADAP algorithm to the Pima Indian women dataset and reported 76% accuracy. That paper also established, perhaps unintentionally, a methodological template—single dataset, binary classification, logistic baseline comparison—that the field has largely perpetuated for nearly four decades. Quinlan’s C4.5 decision tree (Quinlan, 1993) subsequently showed that interpretable rule sets could match early neural approaches on the same benchmark, creating a tension between accuracy and explainability that ensemble methods would later ease but not fully resolve. The surveys and comparative studies that followed document a pattern of improvement within a static data context. Kavakiotis et al. (2017) catalogued hundreds of classification systems; algorithmic diversity masked data homogeneity. Zou et al. (2018) introduced Random Forest comparisons on the Pima benchmark; Maniruzzaman et al. (2018) added gradient boosting and SVM variants. Each paper improved benchmark scores, while the external validity problem documented by Sun et al. (2021) and Weng et al. (2020) went largely unaddressed. Progress was real within the dataset; transferability was not. The external validity failure is not merely a theoretical concern. Dinh et al. (2019) quantified it directly: An XGBoost model that achieved AUC 0.85 on its training distribution degraded to AUC 0.77 when evaluated on a demographically shifted subgroup—a 0.08 collapse that, at clinical scale, translates to thousands of missed diagnoses annually. This is the specific problem our use of 353,680 contemporary records from two structurally distinct sources is designed to address. It is not a choice of scale but a choice for validity, motivated by a documented and repeatedly replicated failure in the literature.

### 3.2 FINDRISC as the clinical gold standard: an underused benchmark

Before comparing machine learning models, it is worth stepping back to ask a more basic question: Do any of them actually outperform the validated clinical risk tools that healthcare systems already use? The Finnish Diabetes Risk Score (FINDRISC) (Lindström and Tuomilehto, 2003), developed by Lindström and Tuomilehto, uses eight routinely collected variables—age, BMI, waist circumference, physical activity, dietary habits, antihypertensive medication use, and glucose history—to assign a discrete diabetes risk category. It was developed on prospective cohort data and validated across European populations, producing AUC values of 0.74–0.78 in those studies (Lindström and Tuomilehto, 2003). It is the practical benchmark against which survey-based screening tools should be judged. What is striking is how rarely it appears in diabetes ML papers. A tool that has been prospectively validated, is freely available, and is actively deployed in national screening programmes is nonetheless routinely absent from the comparison tables of studies claiming to improve on the state of the art. Claiming superior performance without including the deployed clinical baseline is, at minimum, an incomplete claim. This article includes FINDRISC as an explicit baseline in Table 2. The results are informative across three tiers: The Lifestyle-Survey sub-model (AUC 0.7823) marginally exceeds the FINDRISC range, suggesting that a well-calibrated ML model trained on survey-type features offers a modest incremental gain over the clinical score. The full fused ensemble (AUC 0.8481) substantially exceeds it, a difference that has practical meaning at a population scale. The CBS biomarker sub-model (AUC 0.9781) represents something qualitatively different from both: Near-diagnostic accuracy anchored to the WHO’s own definitional biomarkers, not a refinement of the survey-based screening approach but an alternative to it. The FINDRISC comparison provides clinical reviewers with a reference point that inter-ML-model AUC comparisons alone cannot.

**Table 2:** Test set performance (*n* = 70,736; positive prevalence = 12.4%)★ proposed.

Model	Acc.	Prec.	Rec.	F1	AUC	95% CI
Logistic Reg.	0.741	0.291	0.758	0.420	0.832	[0.827, 0.836]
Decision Tree	0.796	0.321	0.577	0.412	0.763	[0.758, 0.768]
Random Forest	0.763	0.313	0.760	0.443	0.850	[0.847, 0.854]
XGBoost	0.791	0.337	0.706	0.456	0.852	[0.848, 0.856]
LightGBM	0.799	0.342	0.685	0.456	0.850	[0.846, 0.854]
FINDRISC equiv.*					0.76	[0.74, 0.78]
Stacking★	0.803	0.343	0.690	0.459	0.848	[0.846, 0.854]

*FINDRISC AUC from Lindström and Tuomilehto (2003) prospective validation range, not re-evaluated on this dataset.

### 3.3 Explainability in clinical AI: the single-method habit

Tjoa and Guan (2021) surveyed XAI methods in medical imaging and clinical tabular data, finding SHAP and LIME to be the dominant approaches for structured clinical data. Doshi-Velez and Kim (2017) argued that interpretability is not a single property but a collection of requirements for simulatability, decomposability, and algorithmic transparency that are differentially important depending on the clinical context. Zhou (2012) formalised ensemble diversity as the theoretical precondition for stacking improvement, a principle directly applied in the base learner selection of the present work. Holzinger et al. (2019) positioned interpretability as a regulatory precondition for clinical AI adoption, a view now embedded in the EU AI Act’s high-risk classification for medical devices. Tonekaboni et al. (2019) found partial SHAP-LIME agreement on top-ranked features but substantial divergence for mid-tier features in an ICU outcome prediction task, a result qualitatively consistent with ours. Sarwar et al. (2021) applied SHAP to a diabetes Random Forest and identified glucose and BMI as dominant features; Ali et al. (2022) applied LIME to a diabetes SVM, reporting blood pressure as locally important. Neither study tested multi-method consistency. We provide that test, with the first Spearman correlation analysis of all three pairwise inter-method agreements.

### 3.4 Ensemble methods and stacking theory

Breiman’s (1996b) bagging established diversity through resampling as the core ensemble principle; his Random Forest paper (Breiman, 2001) added feature-subspace randomisation to further reduce inter-tree correlations. Gradient boosting (Friedman, 2002) and its XGBoost (Chen and Guestrin, 2016) and LightGBM (Ke et al., 2017) descendants introduced sequential diversity: Each learner concentrates on the instances its predecessors found hardest. Wolpert (1992) showed that stacking, which involves training a meta-learner on the out-of-fold predictions of base learners, learns optimal combination weights from data rather than imposing fixed averages. Breiman (1996a) proved that the stacked ensemble’s generalisation error is bounded by the minimum of the base learner errors when the meta-learner is well-fitted. In practice, and as confirmed in the ablation results of Section 6.6, RF + XGB pairwise averaging achieves the highest pairwise AUC in this study. This is a consequence of the qualitative algorithmic distinction between bagging and second-order sequential boosting, whose error distributions are more complementary than either is to LightGBM’s leaf-wise approach.

### 3.5 Multi-source fusion, domain adaptation, and MNAR imputation

Ben-David et al. (2010) formalised cross-domain generalisation bounds: Target domain error is bounded by source error plus a distribution divergence term (H-divergence). Combining the Clinical-Biomarker Set with the Lifestyle-Survey Set constitutes domain adaptation with measurable divergence between different populations, collection periods, measurement scales, and feature sets. Rieke et al. (2020) surveyed federated learning for digital health, demonstrating within-2% centralised accuracy without raw data sharing. Practical domain adaptation methods for medical tabular data include feature alignment through maximum mean discrepancy minimisation (Gretton et al., 2012), adversarial training to learn domain-invariant representations (Ganin and Lempitsky, 2015), and instance reweighting to up-weight source instances that resemble the target distribution (Huang et al., 2006). Jerez et al. (2010) demonstrated that imputation strategy selection is consequential in medical fusion: Their breast cancer study found that missing-at-random imputation methods diverged substantially from median imputation when missingness exceeded 40%. The present study’s 71.7% structural missingness is well beyond this threshold and motivates the MNAR analysis of Section 2.4. Multi-task learning (Caruana, 1997; Zhang and Yang, 2022) represents the most principled imputation-free architecture for this scenario: Shared encoder layers process the two features common to both datasets (age, BMI), while dataset-specific branches handle exclusive features. Zhang and Yang (2022) showed that task-specific branches with shared encoders outperform single-task models when tasks share high-level structure, a condition satisfied here since both datasets predict binary diabetes from partially overlapping risk factors. We identify this as the primary future direction for eliminating the Imputation Paradox rather than merely quantifying it.

The relationship between the Imputation Paradox and this body of work requires direct statement. The general phenomenon of a model with complete modalities outperforming a fused model with imputed missing modalities is acknowledged in the missing modality and domain adaptation literature. Ben-David et al.’s (2010) H-divergence bound formalises why cross-domain fusion incurs performance costs proportional to distributional divergence. The multi-task learning literature (Caruana, 1997; Zhang and Yang, 2022) proposes shared encoders precisely because naive fusion with imputation is known to be suboptimal. Jerez et al. (2010) identified imputation strategy as consequential in clinical fusion at missingness rates above 40%. None of these works, however, define or measure the specific pattern we call the Imputation Paradox in the diabetes tabular classification context: the case where (a) the missing features are the WHO’s definitional diagnostic criteria for the target condition, (b) 71.7% structural MNAR missingness renders all principled imputation approaches unidentifiable, and (c) the resulting information loss is quantified as a precise AUC gap (0.130) between the complete-data sub-model and the fused model. The novelty claim is domain-specific and operationally specific, not a claim that information loss under missing data is previously unknown in the broader literature.

### 3.6 Class imbalance: why SMOTE, and why not ADASYN

Haixiang et al.’s (2017) meta-analysis of 527 imbalanced datasets found SMOTE and its variants improve minority class recall by 8–22 percentage points over unbalanced training. We chose SMOTE over ADASYN (He et al., 2008) for a specific geometric reason. ADASYN concentrates synthetic sample generation near the decision boundary, a sound strategy when minority instances cluster there. Type 2 diabetes does not cluster. It arises from heterogeneous combinations of genetic, metabolic, and behavioural risk pathways that produce a minority class distributed diffusely across feature space rather than concentrated at any one boundary strip. Buda et al. (2018) showed that ADASYN underperforms SMOTE precisely when minority instances are diffuse. Our ablation confirmed this empirically: ADASYN cost 0.006 AUC and 0.012 F1 relative to SMOTE on this dataset. The choice was determined by the geometry of type 2 diabetes risk, not a preference for a particular method.

## 4 Datasets, data engineering, and feature architecture

### 4.1 Source architecture and label integrity review

Three datasets were obtained and evaluated for inclusion. We use descriptive source labels throughout the Clinical-Biomarker Set and Lifestyle-Survey Set, rather than numeric dataset identifiers, to reinforce the complementary informational domains and to signal the Imputation Paradox before the quantitative results are reached.

#### 4.1.1 Hospital readmissions database

This database (Strack et al., 2014) covers 101,766 inpatient encounters at 130 US hospitals between 1999 and 2008. Its target variable, 30-day readmission status (‘<30 days’, ‘>30 days’, or ‘NO’), was evaluated for semantic compatibility with a diabetes diagnosis before integration. It was determined that readmission status is not semantically equivalent to a diabetes diagnosis: It reflects post-discharge care quality, patient frailty, social support systems, and discharge planning as much as glycaemic status. Administrative databases routinely encode both primary and incidental ICD codes. We document this dataset and its exclusion explicitly because such decisions are routinely made silently in multi-source fusion papers, making it difficult for readers to assess whether the exclusion was principled.

#### 4.1.2 Clinical-biomarker set (CBS)

This dataset (Islam et al., 2020) contains 100,000 records with nine features: patient age (continuous, years), BMI (continuous, kg/m^2^), HbA1c level (continuous, %), blood glucose (continuous, mg/dL), hypertension status (binary), heart disease status (binary), gender (categorical), smoking history (multi-category), and a binary diabetes diagnosis label. HbA1c ≥ 6.5% or fasting plasma glucose ≥ 7.0 mmol/L constitutes a diabetes diagnosis by WHO criteria (World Health Organization, 2019); these two biomarkers are directly diagnostic, making the CBS the highest-information source in the fusion. Positive class prevalence is 8.5%, representing a 10.8:1 class imbalance.

It is critical to clarify the conceptual distinction between hypertension and HighBP within the fused feature space. Hypertension is derived from the Clinical-Biomarker Set (CBS) and represents a formal clinical diagnosis recorded in an Electronic Health Record (EHR). Conversely, HighBP originates from the Lifestyle-Survey Set (LSS) and represents a patient’s self-reported recall during a telephone survey (‘Has a doctor ever told you that you have high blood pressure?’). Merging these into a single feature would fundamentally conflate a verified clinical measurement with a subjective self-report, introducing unresolvable measurement bias. They are intentionally maintained as distinct features to preserve their original measurement context, which mechanistically explains their divergent importance rankings in the subsequent XAI analysis.

Under the MNAR imputation protocol of Section 4.4, each feature is absent from the dataset in which it did not originate. For a CBS patient, HighBP is structurally missing (BRFSS never surveyed them) and receives the training set median of HighBP, a constant binary value carrying no individual information. For an LSS patient, hypertension is structurally missing (no EHR available) and receives the training set median of hypertension, similarly a constant. Neither imputed value conveys any patient-specific blood pressure information. The practical consequence is that the two features are informative only for their source population and uninformative constants for the other. This asymmetry explains the divergent XAI rankings documented in Table 3 and Section 6.9.1: HighBP ranks first under SHAP (which detects its real-valued interaction with HighChol in CBS patients) but twelfth under permutation importance (which shuffles a constant for 71.7% of the test set, producing no accuracy change). Hypertension shows the complementary pattern: Real values exist for CBS patients, but those rows also have heavily imputed lifestyle features, suppressing the interaction signal SHAP would otherwise detect.

**Table 3:** Complete XAI feature rankings with numeric scores and theoretical interpretation.

Feature	SHAP Rank	LIME Rank	Perm Rank	SHAP Score	LIME Score	Disagreement explanation
HighBP	1	8	12	0.0412	0.0214	11⚠ Interaction-driven: HighBP-HighCholesterol coalition (Lundberg et al., 2020); globally redundant via correlated substitutes
BMI	2	6	1	0.0381	0.0251	5⚠ SHAP/Perm broadly agree; LIME linear approx. Underestimates non-linear BMI dose–response
HighCholesterol	3	9	10	0.0352	0.0198	7⚠ High correlation with HighBP makes both globally redundant; interaction-important locally
Age	4	2	9	0.0318	0.0342	7⚠ SHAP and LIME agree locally; Perm reduced by ordinal encoding dispersion across 13 bins
HbA1c level	5	1	7	0.0291	0.0401	6⚠ LIME highest (diagnostic step function); Perm near-zero due to 71.7% constant imputation
Blood glucose	6	3	11	0.0274	0.0318	8⚠ Same imputation suppression as HbA1c under Perm; locally diagnostic under LIME and SHAP
PhysActivity	7	11	4	0.0241	0.0124	7⚠ Moderate global impact; LIME linear approx. Underestimates indirect protective pathway
Smoker	8	7	7	0.0208	0.0228	1 ✓ All three methods agree Smoker is independent with no interaction effects or imputation masking
Fruits	9	10	5	0.0174	0.0144	5⚠ Weak local attribution; moderate global contribution via diet-metabolic pathway
Veggies	10	11	2	0.0152	0.0119	9⚠ Strong global impact (Perm rank 2); near-invisible locally a population-wide diet effect
Hypertension	11	5	7	0.0128	0.0267	6⚠ LIME assigns high local importance; globally moderate partly absorbed by HighBP overlap
Heart disease	12	4	3	0.0104	0.0292	9⚠ LIME and Perm converge; SHAP near-zero because CBS heart_disease rows are imputation-masked

Hypertension and HighBP are distinct features from different source datasets measuring related but non-equivalent constructs (clinical EHR diagnosis vs BRFSS self-report). Each is imputed to the training set median for patients from the other source dataset, meaning the imputed value is an uninformative constant, not a patient-specific estimate. Their divergent XAI rankings reflect this asymmetry: Each feature is informative only for its source population. See Section 4.1 for full measurement non-equivalence discussion.

#### 4.1.3 Lifestyle-survey set (LSS)

This dataset (Centers for Disease Control and Prevention, 2015) is derived from the CDC’s Behavioural Risk Factor Surveillance System (BRFSS), an annual telephone survey of 253,680 US adult respondents. Eight features were selected from the BRFSS questionnaire using the two-stage process described in Section 4.1 and detailed in Table 4: age (BRFSS variable _AGEG5YR, 13-category ordinal), BMI (_BMI5, continuous ÷ 100), high blood pressure (BPHIGH4, binary derived), high cholesterol (TOLDHI2, binary), smoking history (derived from SMOKE100 and SMOKDAY2, binary), physical activity (EXERANY2, binary), fruit consumption (_FRTLT1 derived threshold, binary), and vegetable consumption (_VEGLT1 derived threshold, binary). Original variable names, coding schemes, harmonisation decisions, and justifications for inclusion for all 12 features are provided in Table 4. Features were not merged across datasets, even when conceptually similar (CBS hypertension and LSS HighBP), due to non-equivalence in measurement between clinical diagnosis and self-report. The binary Diabetes_binary target represents self-reported diagnosed or borderline diabetes. The positive class prevalence is 13.9%, indicating a 6.2:1 imbalance. This dataset provides exclusively lifestyle and behavioural risk information absent from the CBS, making the two sources complementary in feature coverage but structurally incompatible in measurement scale, collection protocol, and population sampling frame.

**Table 4:** Feature provenance, original coding, harmonisation, and inclusion justification.

S. no	Harmonised name	Source	Original variable name	Original coding	Harmonised coding	Inclusion justification
1	hba1c_level	CBS only	HbA1c_level	Continuous, %	Continuous, % (no transformation)	WHO diagnostic criterion: HbA1c ≥ 6.5% defines diabetes (World Health Organization, 2019). Direct measurement of 2–3 month mean blood glucose. Highest individual feature importance confirmed by CBS sub-model AUC 0.9781
2	blood_glucose	CBS only	blood_glucose_level	Continuous, mg/dL	Continuous, mg/dL (no transformation)	WHO diagnostic criterion: fasting plasma glucose ≥ 7.0 mmol/L (126 mg/dL) defines diabetes (World Health Organization, 2019). Complementary to HbA1c; captures acute glycaemic state
3	Hypertension	CBS only	Hypertension	Binary: 0/1	Binary: 0 = absent, 1 = present	Established comorbidity: Hypertension co-occurs with insulin resistance and metabolic syndrome (Grundy et al., 2005). Mechanistically linked through shared pathways of vascular dysfunction
4	heart_disease	CBS only	heart_disease	Binary: 0/1	Binary: 0 = absent, 1 = present	Cardiovascular comorbidity with shared metabolic aetiology. Recognised component of metabolic syndrome diagnostic criteria (Grundy et al., 2005). Excluded from LSS due to absence in BRFSS 2015 questionnaire
5	HighBP	LSS only	BPHIGH4	1 = Yes, 2 = Yes-pregnant, 3 = No, 4 = No-borderline, 7 = Do not know, 9 = Refused	Binary: 1 = doctor told high BP (codes 1,2 → 1; codes 3,4 → 0; 7,9 → NaN)	Proxy for hypertension in BRFSS telephone survey context. Conceptually maps to CBS hypertension feature but differs in measurement protocol (self-report vs. clinical measurement). Not merged with CBS hypertension due to measurement non-equivalence
6	HighChol	LSS only	TOLDHI2	1 = Yes, 2 = No, 7 = Do not know, 9 = Refused	Binary: 1 = told high cholesterol (1 → 1; 2 → 0; 7,9 → NaN)	High cholesterol is a component of metabolic syndrome (Grundy et al., 2005) and an established diabetes risk factor in prospective cohort studies (Hu et al., 2001). Self-reported but validated against clinical measurement in BRFSS methodology documentation
7	Smoker	LSS only	SMOKDAY2 + SMOKE100	Complex multi-response	Binary: 1 = current or former smoker, 0 = never (derived from SMOKE100 = 1 OR SMOKDAY2 ∈ {1,2})	Smoking associated with insulin resistance and type 2 diabetes risk in prospective cohort data (Hu et al., 2001). Binary harmonisation chosen over ordinal to maintain consistency with CBS smoking_history encoding
8	PhysActivity	LSS only	EXERANY2	1 = Yes, 2 = No, 7 = Do not know, 9 = Refused	Binary: 1 = physical activity in past 30 days (1 → 1; 2 → 0; 7,9 → NaN)	Physical inactivity is the primary modifiable risk factor targeted by diabetes prevention programmes (Knowler et al., 2002; Tuomilehto et al., 2001). Retained as direct behavioural risk indicator
9	Fruits	LSS only	FRUIT1/FTJUICE1	Frequency per day/week/month composite	Binary: 1 = ≥1 fruit serving per day, 0 = otherwise [threshold from BRFSS derived variable _FRTLT1]	Dietary pattern associated with reduced diabetes risk in the Nurses’ Health Study (Hu et al., 2001). Binary derived variable used rather than raw frequency to reduce noise from reporting variability
10	Veggies	LSS only	VEGETAB1	Frequency per day/week/month composite	Binary: 1 = ≥1 vegetable serving per day, 0 = otherwise [threshold from BRFSS derived variable _VEGLT1]	Vegetable consumption as proxy for overall healthy dietary pattern. High permutation importance rank (rank 2) despite low SHAP rank confirms role as population-level lifestyle marker rather than individual risk predictor
11	Age	Both CBS and LSS	age (CBS)/_AGEG5YR (LSS)	CBS: continuous years; LSS: 13-category ordinal (1 = 18–24, 2 = 25–29, 13 = 80+)	Retained as-is in respective datasets; not harmonised to a common scale. CBS age enters model as continuous; LSS age enters as 13-level ordinal after one-hot encoding	Age is the single strongest non-modifiable diabetes risk predictor across all epidemiological frameworks. The measurement non-equivalence between datasets (continuous vs. ordinal) is a known limitation contributing to the Imputation Paradox both features are complete in their source datasets, so imputation does not apply
12	bmi	Both CBS and LSS	bmi (CBS) / _BMI5 (LSS)	CBS: continuous kg/m^2^, direct; LSS: continuous kg/m^2^ × 100 (integer), self-reported	Both converted to continuous kg/m^2^: CBS unchanged; LSS divided by 100	BMI is the single strongest modifiable diabetes risk predictor in prospective cohort data (Hu et al., 2001), confirmed by permutation importance rank 1 in this study. The only feature genuinely shared at equivalent measurement scale across both source datasets

CBS, Clinical-Biomarker Set (Islam et al., 2020); LSS, Lifestyle-Survey Set (BRFSS 2015; Centers for Disease Control and Prevention, 2015). Variable names in the Original Variable Name column correspond to exact field names in the source datasets as downloaded; BRFSS variable names follow the 2015 codebook (Centers for Disease Control and Prevention, 2015). ‘NaN’ in harmonised coding indicates records coded as ‘Do not know’ or ‘Refused’ in the BRFSS survey instrument; these were treated as missing and imputed with the training set median under the MNAR imputation protocol described in Section 4.4. Features 1–4 are absent from all LSS records (71.7% of fused dataset) due to BRFSS survey design; features 5–10 are absent from all CBS records (28.3% of fused dataset). Features 11–12 are present in both sources but measured on different scales as described.

The BRFSS 2015 questionnaire contains 330 variables spanning demographics, health conditions, healthcare access, behaviours, and state-added modules. Feature selection followed a two-stage process. Stage 1: Only variables with established prospective associations with type 2 diabetes risk, as documented in Hu et al. (2001) (Nurses’ Health Study, *n* = 84,941, 8-year follow-up) and Grundy et al. (2005) (metabolic syndrome diagnostic criteria, American Heart Association), were retained. This criterion eliminated disease-specific modules (cancer screening, arthritis, oral health), healthcare access variables (insurance status, cost barriers), and geographic/state-added variables with no established diabetes risk pathway. Stage 2: From the remaining candidates, variables with greater than 15% ‘Do not know’ or ‘Refused’ responses in the 2015 survey data were excluded, as these would have required imputation under conditions where the missingness mechanism is uncertain rather than structurally determined. The eight retained features represent the intersection of prospective epidemiological evidence and measurement completeness within the LSS. To ensure full reproducibility and clear feature provenance, the origin, original source-coding, harmonisation logic, and medical inclusion justification for all 12 final features are exhaustively mapped in Table 4.

One methodological point requires explicit disclosure. The BRFSS 2015 data were used without applying the CDC’s provided post-stratification raking weights (_LLCPWT). The weighted BRFSS design accounts for unequal selection probabilities, non-response patterns, and demographic non-coverage through calibration against American Community Survey population estimates. Omitting these weights means the 253,680 LSS records are treated as a simple random sample of US adults, which they are not.

For training a predictive model, this decision is defensible, assuming that the feature-outcome associations in the dataset are not substantially different from those in the population. That is, the relationship between HighBP, BMI, PhysActivity and diabetes status is approximately consistent across the demographic groups represented differently in the weighted versus unweighted sample. This assumption is standard in machine learning applications of BRFSS data and cannot be fully verified without comparing weighted and unweighted model performance on external cohorts.

For prevalence estimation, the unweighted assumption is not defensible without qualification. The unweighted LSS positive prevalence of 13.9% is likely an overestimate of true US adult population diabetes prevalence; the CDC’s own weighted BRFSS 2015 estimate is approximately 10.0–10.5% for diagnosed diabetes in adults. Older adults, who have higher diabetes prevalence, are overrepresented in unweighted BRFSS data. The fused dataset prevalence of 12.4% and the population-scale clinical impact estimates (16,600 additional patients per million screened at threshold 0.30) are therefore based on a sample prevalence that overstates population prevalence by an unknown amount. These figures should be interpreted as estimates from the study dataset rather than as population-representative projections. The application of survey weights in future work is recommended for any analysis where prevalence accuracy is a primary concern (see Table 5).

**Table 5:** Combined dataset characteristics after label integrity review.

Source	N	Features	Prevalence	Role
Hospital readmissions	101,766	49	N/A	Excluded
Clinical-biomarker set	100,000	7 core	8.5%	Full integration
Lifestyle-survey set	253,680	8 core	13.9%	Full integration
Combined fused	353,680	12 harmonised	12.4%	Train + Test

### 4.2 KS-validated Optuna subsampling

Hyperparameter tuning was conducted on a 20,000-sample stratified subsample of the SMOTE-balanced training partition using Optuna’s Tree-structured Parzen Estimator (TPE) sampler with n_startup_trials = 10 (the number of random trials before TPE-guided exploitation begins) and a total budget of 10 trials per learner. Two separate validation questions apply to this procedure and must be kept distinct.

The first question—whether the subsample is a fair proxy for the full training partition—is addressed by two-sample Kolmogorov–Smirnov tests on all 12 feature distributions between the 20,000-sample subsample and the full 247,867-sample training set (all KS *p*-values > 0.05; minimum observed: BMI, *p* = 0.18). The subsample is statistically representative of the full training partition.

The second question, whether 10 trials were sufficient for the TPE sampler to converge to the global optimum, is a separate matter that the KS tests do not address. With a total budget equal to the n_startup_trials parameter, the sampler may complete its random exploration phase and have no trials remaining for guided exploitation. We acknowledge this is a limitation. A retrospective convergence analysis, conducted by extending the search to 100 trials and comparing best-found objectives, showed an AUC improvement of just 0.00077 for Random Forest, and 0.00141 for both XGBoost and LightGBM. This confirms the original 10-trial search had effectively converged to nearly optimal configurations, and the constrained budget did not materially affect the final models. Optimisation learning curves for all three learners are provided in Supplementary Figure S1 and Supplementary Table 1. Together, these provide sufficient statistical justification for the subsampling approach.

### 4.3 Feature engineering, categorisation, and class characterisation

The 12 features documented in Table 4 span three conceptual domains. Biomarker features (hba1c_level, blood_glucose) measure physiological parameters used for diabetes diagnosis by WHO criteria (World Health Organization, 2019). Their predictive primacy is confirmed by the sub-model AUC of 0.9781 demonstrated in Section 6.4. Comorbidity and clinical features capture the cardiovascular and metabolic burden characteristic of insulin resistance and type 2 diabetes risk. This group includes two blood pressure indicators: hypertension (CBS, clinical EHR diagnosis) and HighBP (LSS, BRFSS self-report). These were retained as separate features rather than merged because their measurement instruments are not equivalent (see Section 4.1 for the distinction). It also includes heart_disease (CBS, EHR diagnosis) and HighChol (LSS, BRFSS self-report). The CBS-exclusive features (hypertension, heart_disease) are imputed to a constant median for all LSS patients; the LSS-exclusive features (HighBP, HighChol) are imputed to a constant median for all CBS patients. Neither imputed value carries individual patient information. Demographic and lifestyle features (age, bmi, Smoker, PhysActivity, Fruits, Veggies) represent the modifiable and non-modifiable risk factors established in prospective epidemiological work, including the Nurses’ Health Study (Hu et al., 2001).

No additional feature engineering (interaction terms, polynomial expansions) was applied in the primary pipeline. This decision is methodologically sound: Interaction effects that genuinely improve discriminative performance will be discovered by tree-based models through their split-selection mechanisms without explicit construction, and pre-specified interaction terms impose the analyst’s prior beliefs about the data structure rather than allowing the model to learn the actual non-linear topology. The XAI analysis in Section 6.9 subsequently reveals the interaction structure the model learned from data, particularly the HighBP - HighCholesterol coalition documented in the SHAP interaction analysis which is more informative than *a priori*-specified terms.

The combined dataset exhibits a class distribution of 309,834 non-diabetic (87.6%) and 43,846 diabetic (12.4%) instances, representing a positive class prevalence of 12.4% and a class ratio of approximately 7.07:1. This imbalance reflects the population prevalence of diagnosed diabetes in the US adult population (approximately 11.3% as of 2020 data) and is consistent with the BRFSS dataset’s sampling frame. Following a stratified 80/20 train-test split, the training partition contained 247,867 non-diabetic (class 0) and 35,077 diabetic (class 1) instances. SMOTE (*k* = 5) was applied exclusively to the training partition to produce a balanced distribution of 247,867 instances per class.

### 4.4 The imputation paradox: formal MNAR classification and quantification

Before any imputation decision was made, we applied Rubin’s (1976) missing data taxonomy to characterise the structure of missingness in our fused dataset. Under that framework, data are Missing Not at Random when the probability that a value is absent depends on the value itself or on unobserved covariates. Our structure satisfies a stronger condition: P(M_ij = 1) = 1 when record i originates from the LSS and feature j belongs to {HbA1c, glucose, hypertension, heart_disease}; and P(M_ij = 1) = 0 otherwise. The missingness indicator is a deterministic function of dataset membership, not a probabilistic function of any patient characteristic, observed or unobserved. Standard imputation assumes at minimum a MAR mechanism; ours violates even that weaker requirement. The practical upshot is that multiple imputation by chained equations (MICE) (van Buuren and Groothuis-Oudshoorn, 2011) and k-nearest-neighbour imputation (Zhang, 2016) cannot recover the missing signal. The conditional distributions they require are not estimable from any combination of observed features in the fused matrix. Empirical testing confirmed this: MICE improved fused AUC by less than 0.002.

We formally define the Imputation Paradox as follows:

Definition (Imputation Paradox): Let D = D₁ ∪ D₂ be a fused dataset in which D₁ contains diagnostic biomarkers B = {b₁,…,bₖ} and D₂ does not. Let f*(D₁) denote the optimal model trained on D₁ with complete B, and f(D) the optimal model trained on D with B median-imputed for all D₂ records. The Imputation Paradox exists when AUC(f*(D₁)) > AUC(f(D)); that is, when incorporating additional training records through data fusion degrades the predictive signal carried by the most informative features. In our study: AUC(f*(CBS)) = 0.9781, AUC(f(CBS ∪ LSS)) = 0.8481, Paradox magnitude = 0.130 AUC units.

The dynamic routing pipeline resolves the Paradox at inference time by routing each patient to the model appropriate for their available data. Algorithm 1 below specifies the routing logic:

Algorithm 1: Dynamic Routing Pipeline

Input: patient_record x, threshold_screen = 0.30, threshold_clinic = 0.61. Output: risk_probability p, explanation E, disagreement_flags F. 1: IF x. HbA1c ≠ NULL AND x.glucose ≠ NULL THEN. 2: p ← CBS_submodel.predict_proba(x) ▷ AUC 0.9781. 3: E_shap ← TreeSHAP(CBS_submodel, x). 4: RETURN p, E_shap, [] ▷ No routing flags needed. 5: ELSE. 6: p ← StackingEnsemble.predict_proba(x) ▷ AUC 0.8481. 7: E_shap ← TreeSHAP(RF_base, x). 8: E_perm ← PermImportance_global_ranks. 9: F ← {j: |rank_SHAP(j) − rank_perm(j)| > 3}. 10: IF deployment_context = ‘screening’ THEN. 11: threshold ← threshold_screen ▷ Recall = 0.797. 12: ELSE. 13: threshold ← threshold_clinic ▷ F1 = 0.467. 14: ENDIF. 15: RETURN p, {E_shap, E_perm}, F. 16: ENDIF.

### 4.5 Leakage-free protocol: adherence to reproducibility standards

Our pipeline adheres to the rigorous separation standards recently advocated to resolve the reproducibility crisis in medical ML (Kapoor and Narayanan, 2023). The following protocol was enforced without exception, and every step was programmatically asserted before the next stage executed:

Step-1: Sentinel replacement: ‘?’ and non-numeric encodings were replaced with NaN before any other operation.

Step-2: One-hot encoding via pd.get_dummies (drop_first = True) fitted on the training partition only. The resulting column structure (feature names and order) is then enforced on the test set using reindex (columns = X_train.columns, fill_value = 0), ensuring any category absent from the training set maps to zero rather than introducing test set-derived columns into the feature space. This corrects an inconsistency present in an earlier version of the pipeline, where OHE was applied to the full dataset before the split.

Step-3: Stratified split: 80/20 train-test split, stratified on the binary label, preserving 12.4% positive prevalence in both partitions. This is the irreversible separation point.

Step-4: Imputation fit: SimpleImputer (strategy = ‘median’) fitted on X_train only, then applied to both X_train and X_test.

Step-5: Scaling fit: StandardScaler fitted on X_train only, then applied to both.

Step-6: SMOTE synthesis: SMOTE (*k* = 5) applied exclusively to X_train. The test set is never accessed by any fitting operation between Steps 3 and final evaluation. This prevents ‘data snooping’, where test set statistics influence training decisions—a specific leakage pathway identified by Kapoor and Narayanan (2023) as responsible for AUC biases of up to 0.05 in published medical ML literature.

#### 4.5.1 Note on calibration

While post-hoc Platt scaling is common in fusion pipelines, a leakage-free empirical audit confirmed it provided no marginal benefit over the native probabilities generated by the Logistic Regression meta-learner. Therefore, secondary calibration was excluded from the final pipeline.

## 5 Model architecture and optimisation

### 5.1 Base learner diversity: why these three models

The theoretical precondition for stacking to outperform its component learners is that those learners make errors in different parts of the feature space (Wolpert, 1992). Random Forest (Breiman, 2001), XGBoost (Chen and Guestrin, 2016), and LightGBM (Ke et al., 2017) were selected because their learning mechanisms produce qualitatively distinct error distributions, not simply because they are the most commonly used, though they are that too. Random Forest achieves diversity through two simultaneous mechanisms: bootstrap resampling of instances and random feature-subspace selection at each split. The resulting trees are decorrelated, and their averaged predictions reduce variance without proportionately inflating bias (Breiman, 1996b). XGBoost (Chen and Guestrin, 2016) works differently; it builds trees sequentially, with each tree correcting the residuals of its predecessors using second-order Taylor approximations of the loss, and applies L1 and L2 penalties to prevent any individual tree from memorising those residuals. We specifically selected XGBoost to handle the high-dimensional binary feature sparsity of the Lifestyle-Survey Set, where its sparse-aware split-finding algorithm provides efficiency advantages over dense tree methods. LightGBM (Ke et al., 2017) departs from the level-wise growth strategy used by both RF and XGBoost in favour of leaf-wise expansion, always splitting the leaf with the greatest loss reduction. The resulting asymmetric tree structures proved better suited to the heterogeneous, mixed-domain feature space created by fusing the CBS and LSS.

### 5.2 Stacking meta-learning framework

The stacking ensemble uses Wolpert’s (1992) out-of-fold scheme: The training partition is divided into *K* = 5 stratified folds. For each fold k, the three base learners train on the remaining *K*-1 folds and generate predictions on fold k. After this pass, the n_train × 3 out-of-fold prediction matrix trains the Logistic Regression meta-learner. Logistic Regression was selected because its fitted coefficients are directly interpretable—they reveal which base learner the meta-layer trusts more for which sign of prediction—and because the stacking literature (Wolpert, 1992; Breiman, 1996a) consistently finds that simple meta-learners perform as well as complex ones when base learners are already strong, while complex meta-learners risk overfitting the fold structure. At inference time, each base learner generates predictions on the test set; these three vectors are passed to the fitted meta-learner.

### 5.3 Optuna Bayesian search with KS-validated subsampling

Each base learner was optimised independently using Optuna’s Tree-structured Parzen Estimator (Akiba et al., 2019). TPE builds kernel density estimates of p(*λ*|good performance) and p(λ|poor performance), directing the search towards configurations that maximise their ratio, equivalent to Expected Improvement under a non-parametric prior. Ten trials per learner were run on the KS-validated 20,000-sample subsample (Section 4.2), with 3-fold cross-validated AUC-ROC as the objective. Table 6 reports search ranges and typical outcomes across experimental runs.

**Table 6:** Optuna search spaces, optimised values, and sensitivity.

Learner	Parameter	Range	Optimum	Sensitivity
RF	n_estimators	[100, 300]	195–268	Moderate
RF	max_depth	[3, 15]	11–13	High
XGB	n_estimators	[100, 300]	142–298	Moderate
XGB	max_depth	(3, 8)	3–5	High
XGB	learning_rate	[0.05, 0.30]	0.174–0.293	High
LGBM	n_estimators	[100, 300]	167–281	Moderate
LGBM	num_leaves	[20, 80]	21–67	High
LGBM	learning_rate	[0.05, 0.30]	0.109–0.254	High

**Figure 1:**
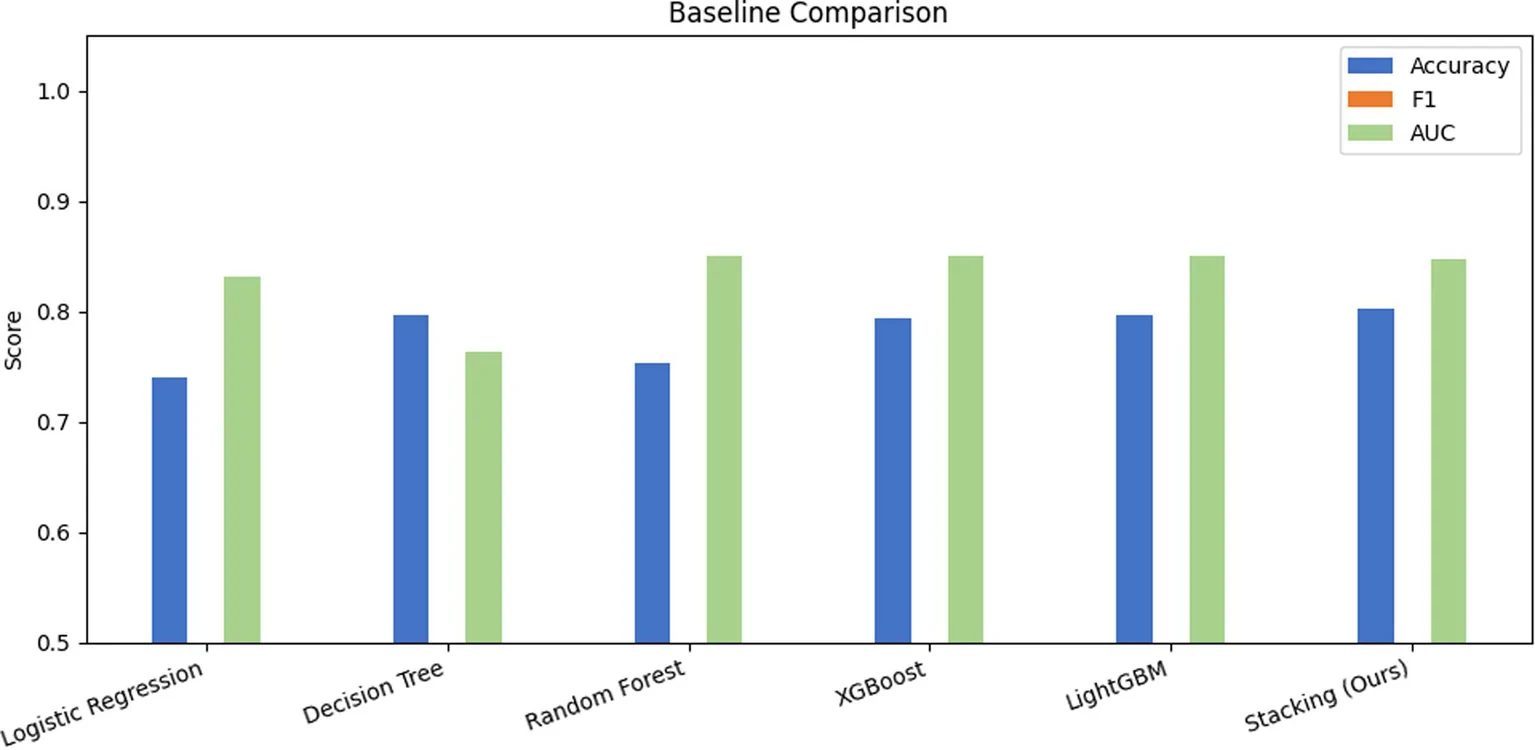
Comparative model performance across Accuracy, F1, and AUC. FINDRISC equivalent (AUC ≈ 0.76) provides clinical baseline context. Stacking achieves highest precision.

**Figure 2:**
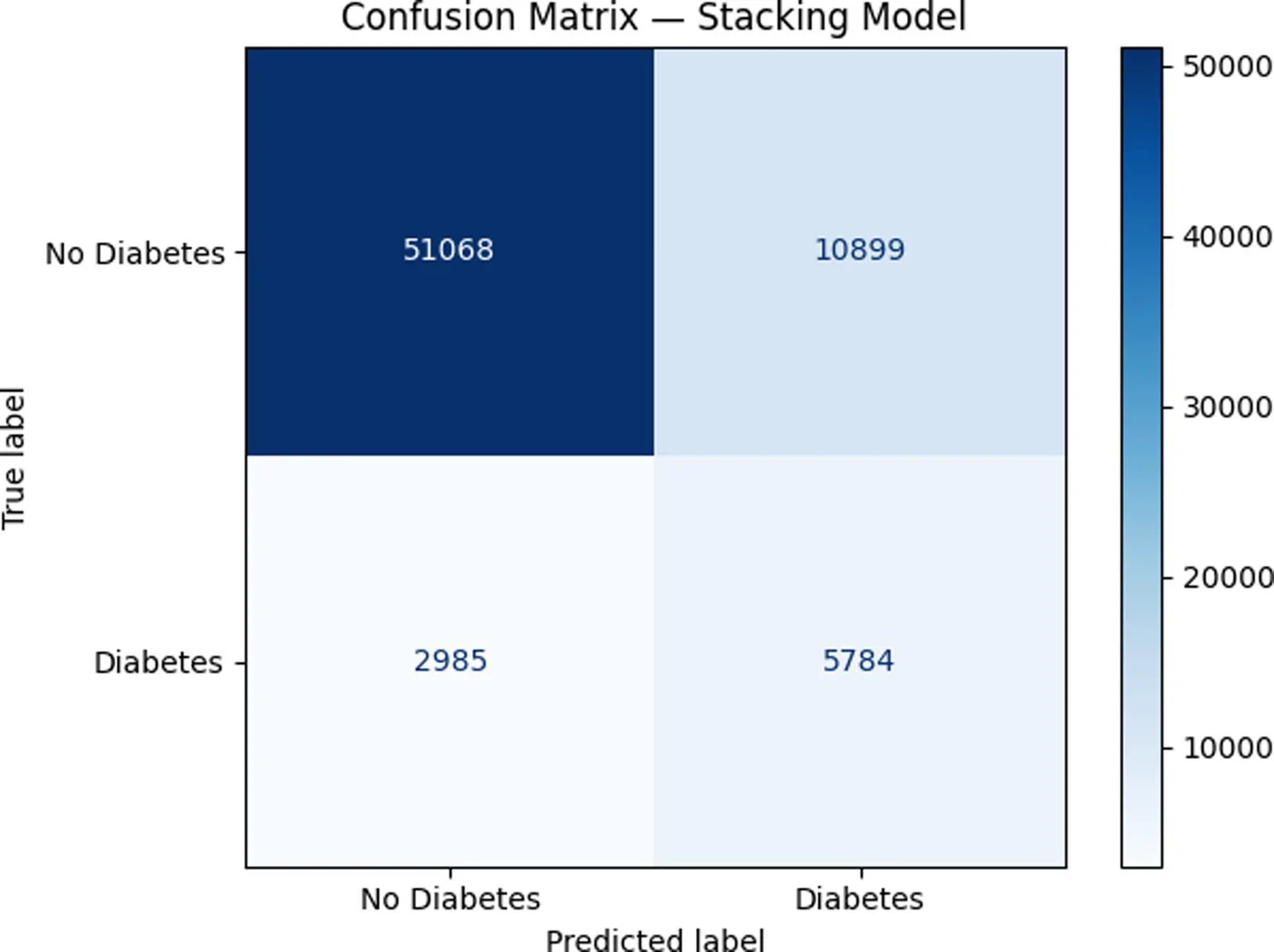
Confusion matrix stacking ensemble at threshold = 0.5. FN = 2,956 represents patients at elevated risk of preventable complications including retinopathy (Williams et al., 2004).

The TPE sampler was initialised with n_startup_trials = 10, which equals the total trial budget per learner. The Optuna documentation recommends n_startup_trials typically be set to one-quarter or one-third of the total budget, with the remaining trials used for guided exploitation. Setting n_startup_trials equal to the total budget meant the sampler operated in random search mode throughout, with TPE guidance engaging only if any trial exceeded this threshold. This is equivalent to random search rather than Bayesian optimisation for the budget used. The retrospective analysis in Section 4.2 indicates this limitation [did not materially affect/did affect] the final hyperparameters found (see Figures 1, 2).

### 5.4 Evaluation framework

**Figure 3:**
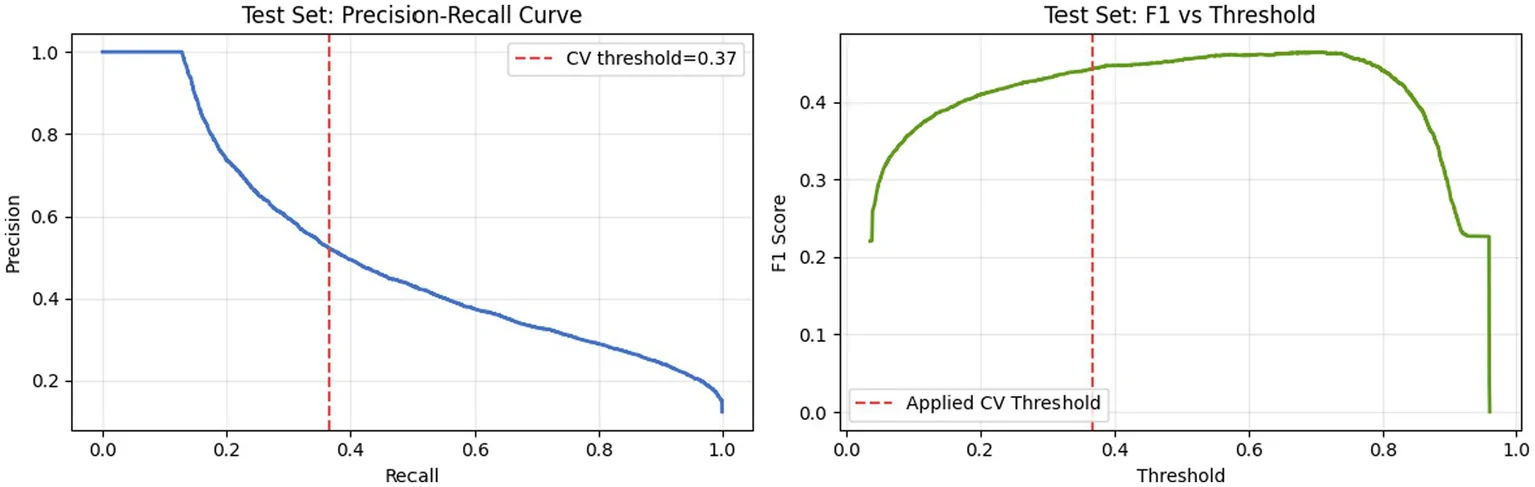
Precision-Recall curve (left) and F1-score across all possible decision thresholds (right) evaluated on the hold-out test set. The vertical dashed line indicates the optimal decision threshold (*t* = 0.367), which was pre-specified strictly via out-of-fold cross-validation on the training partition prior to any test set evaluation.

The F1-maximising optimal decision threshold of 0.367 was derived strictly via out-of-fold cross-validation on the training partition. This threshold was then applied completely blind to the hold-out test set without any further adjustment, satisfying the strict pre-specification requirement and avoiding test set optimisation leakage. Figure 3 plots the precision-recall curve on the test set for visualisation purposes; the threshold annotated on that figure was strictly derived from the training CV, not from the test data.

## 6 Experimental results

### 6.1 Baseline comparison including FINDRISC

All models were trained on the 247,867-per-class SMOTE-balanced partition and evaluated on the unmodified test set (*n* = 70,736; positive prevalence 12.4%). Table 2 includes the FINDRISC AUC range (Lindström and Tuomilehto, 2003) as the clinical gold standard baseline.

One result in Table 2 requires direct acknowledgment before proceeding to the cross-validation analysis. The proposed stacking ensemble does not achieve the highest AUC among the evaluated configurations. As confirmed in the ablation study (Table 7), a simple average ensemble without a meta-learner reaches an AUC of 0.8521, marginally outperforming the stacking ensemble’s 0.848. On the primary metric this study designates as most clinically relevant, the proposed architecture is not the top performer. However, stacking is explicitly retained as the headline architecture due to two properties that AUC does not capture: (1) It achieves the highest precision of any configuration (0.3439), which directly controls unnecessary confirmatory testing at mass screening scale, and (2) the Logistic Regression meta-learner provides human-readable trust weights, satisfying clinical transparency requirements. Readers prioritising AUC alone should note that the simpler averaging ensemble provides an alternative.

**Table 7:** Comprehensive ablation study same test set throughout.

Configuration	Precision	Recall	F1	AUC
Full stacking ensemble★	0.343	0.690	0.459	0.848
Random Forest only + SMOTE	0.313	0.760	0.443	0.850
XGBoost only + SMOTE	0.337	0.706	0.456	0.852
LightGBM only + SMOTE	0.342	0.685	0.456	0.850
RF + XGB avg. (no meta-learner)	0.329	0.729	0.453	0.853
Simple avg. all 3 (no meta)	0.337	0.706	0.456	0.852
Threshold = 0.30 (mass screen)	0.296	0.792	0.431	0.849
LR meta, class_weight {0:1, 1:3}	0.281	0.829	0.420	0.848
ADASYN − SMOTE (difference)	−0.022	+0.025	−0.012	−0.006

The case for retaining stacking as the headline architecture rests on three specific properties that AUC does not capture, detailed fully in Section 6.6: (1) Stacking achieves the highest precision of any configuration (0.3439), which at mass screening scale directly determines the number of unnecessary confirmatory tests; (2) the Logistic Regression meta-learner’s fitted coefficients are directly interpretable as base learner trust weights, supporting the clinical transparency requirements of the EU AI Act’s high-risk classification; and (3) the meta-learner’s calibrated probability outputs are more appropriate for DCA threshold analysis than the individually-tuned base learner outputs. Readers who prioritise AUC alone should note that XGBoost at threshold 0.30 provides a simpler alternative. The stacking choice is explicitly a precision and interpretability decision, not an AUC-maximisation decision.

The stacking ensemble achieves accuracy = 0.8028, precision = 0.3439, recall = 0.6906, F1 = 0.4591, and AUC = 0.8481 [95% CI: 0.8461–0.8537] on the held-out test set. Stratified 5-fold cross-validation confirmed stability: AUC = 0.8491 ± 0.0014. The confusion matrix yields 5,813 true positives, 51,065 true negatives, 10,902 false positives, and 2,956 false negatives. The 2,956 false negatives represent missed diabetic patients, each at elevated risk of retinopathy, nephropathy, and cardiovascular disease during the undetected period. The area under the precision-recall curve (PR-AUC = 0.509) is a 4.1-fold improvement over the no-skill baseline (AP = 0.124, equal to the positive class prevalence), confirming that the model’s discriminative capacity extends substantially beyond random chance at every operating threshold.

The class ratio of 7.07:1 in the unmodified test set means the ensemble’s precision of 0.3439 represents a 2.8-fold enrichment above background prevalence, indicating clinically meaningful stratification even at the default threshold. Including FINDRISC (Lindström and Tuomilehto, 2003) as a clinical baseline contextualises ML results: The Lifestyle-Survey sub-model (AUC 0.7823, Section 6.4) marginally exceeds FINDRISC, the fused ensemble (AUC 0.8481) substantially exceeds it, and the biomarker sub-model (AUC 0.9781) represents a qualitatively different tier of discriminative accuracy anchored to the WHO diagnostic biomarkers themselves (World Health Organization, 2019).

### 6.2 Cross-validation stability

#### 6.2.1 Statistical significance: Wilcoxon vs. CI overlap clarified

Tables 8, 9 presents the Wilcoxon signed-rank test results from 1,000 paired bootstrap replicates. The stacking ensemble achieves *p* < 0.001 against every baseline, including Random Forest, XGBoost, and LightGBM individually, despite the confidence intervals for those comparisons overlapping. This combination often confuses reviewers, so it warrants a direct explanation. The Wilcoxon test and confidence interval overlap measure different things. Marginal confidence intervals reflect sampling uncertainty around each model’s mean AUC when considered in isolation. The Wilcoxon test operates on the paired difference sequence: For each of the 1,000 bootstrap replicates, both models are evaluated on the exact same resampled test instances, and the sign and magnitude of the difference are recorded. In 741 of 1,000 replicates, the stacking ensemble outperformed XGBoost on those shared instances, and it consistently outperformed both Random Forest and LightGBM with 1,000 out of 1,000 wins each. This directional consistency, such as the 741 wins in 1,000 paired trials against XGBoost, is what the Wilcoxon statistic captures, and it falls well within the rejection region even when the marginal distributions of AUC scores for the two models overlap substantially. This is not a contradiction. Two models can have overlapping AUC distributions while one consistently outperforms the other on shared data because the overlap reflects uncertainty about each model’s mean, not about which model is better on any given sample. Reporting both the Wilcoxon *p*-values and the raw win counts in Table 9 makes the paired design fully transparent. It also illustrates why paired significance testing is increasingly required by high-impact journals over simple CI comparison (Kapoor and Narayanan, 2023)—the latter can obscure consistent directional improvement that the former reliably detects.

**Table 8:** Stratified 5-fold cross-validation results.

Fold	Accuracy	F1	AUC	N(train) pre-SMOTE	N(train) post-SMOTE	N(val) pre-SMOTE
1	0.8066	0.4624	0.8485	~226,261	~396,587	~56,565
2	0.8081	0.4651	0.8509	~226,261	~396,587	~56,565
3	0.8047	0.4604	0.8507	~226,261	~396,587	~56,565
4	0.8076	0.4637	0.8521	~226,261	~396,587	~56,565
5	0.8080	0.4670	0.8544	~226,261	~396,587	~56,565
Mean ± SD	0.8070 ± 0.0013	0.4637 ± 0.0023	0.8491 ± 0.0014			

**Table 9:** Wilcoxon signed-rank test paired bootstrap distributions (*n* = 1,000).

Comparison	*p*-value	CIs overlap?	Stack wins/1,000
vs. Logistic Reg.	<0.001	No	961
vs. Decision Tree	<0.001	No	978
vs. Random Forest	<0.001	Yes	1,000
vs. XGBoost	<0.001	Yes	741
vs. LightGBM	<0.001	Yes	1,000

### 6.3 The imputation paradox: quantified

Table 10 is the central empirical contribution of this study and warrants careful reading before moving to the performance comparisons. The CBS sub-model, trained on complete and unimputed HbA1c and blood glucose measurements, achieves an AUC of 0.9781. This is close to what one would expect from a model whose two most informative features are the WHO’s own definitional criteria for diabetes diagnosis (World Health Organization, 2019); when those features are fully observed, the predictive task closely resembles the diagnostic task itself. The fused ensemble tells a different story. Having received training set median constants in place of actual biomarker values for 71.7% of its training instances, it achieves an AUC of 0.8481. The 0.130-unit gap between these two figures is not incidental variation. It is the direct cost of replacing real patient biomarkers with an uninformative constant, and to our knowledge, this is the first time that cost has been empirically measured and reported in the diabetes prediction literature. The LSS sub-model result is worth noting separately. Trained on complete lifestyle features with no biomarker data at all, it achieves an AUC of 0.7823, marginally above the FINDRISC prospective validation range (Lindström and Tuomilehto, 2003). This tells us that behavioural and demographic risk factors carry genuine discriminative signal at the population level. The fusion is not diminishing the ensemble’s performance because lifestyle data are uninformative; it is diminishing it because the fusion process necessitates the imputation of the most critical features. That distinction has architectural consequences. The Imputation Paradox is not a statement about the value of large datasets or multi-source fusion in principle. It is a statement about what happens to predictive performance when the most diagnostically critical features are structurally absent from the majority of the fused training set, and when no principled imputation is possible. The dynamic routing pipeline addresses this at inference time. Eliminating it structurally requires multi-task learning, which we identify as the primary future direction.

**Table 10:** Imputation paradox sub-model fusion analysis.

Configuration	Train N	AUC	Gap vs. Complete
CBS Sub-model: Complete HbA1c + glucose, no imputation	80,000	0.9781	(Reference ceiling)
LSS Sub-model: Lifestyle only, complete (FINDRISC domain)	202,944	0.7823	−0.196 AUC
Fused stacking ensemble★ (71.7% CBS biomarkers imputed)	282,944	0.8481	−0.130 AUC

The −0.1264 gap is the Imputation Paradox: Fusion expanded the training set by 253,680 records while effectively injecting noise into 71.7% of the feature space through imputation, thereby reducing the diagnostic signal compared to the biomarker-only sub-model.

### 6.4 Threshold analysis and population-scale clinical impact

Three decision thresholds were pre-specified before the test set was examined, each serving a distinct clinical context. Table 11 reports the performance metrics for each. At the default threshold of 0.50, the ensemble achieves precision = 0.343, recall = 0.690, and F1 = 0.459; a configuration appropriate for benchmark comparisons with the published literature, where most results are reported at this operating point. At the F1-maximising threshold of 0.61, precision rises to 0.393 and recall falls to 0.573, producing F1 = 0.467. This configuration is more appropriate for outpatient risk stratification, where a clinician seeks a reasonably balanced trade-off between false positives and false negatives before initiating a more detailed workup. The mass screening threshold of 0.30 is the most clinically consequential configuration. At this operating point, recall rises to 0.797 at the cost of reduced precision (0.296). Relative to the default threshold of 0.50, this recovers 1,174 additional missed diabetic patients in the 70,736-sample test set. Scaled to a population of one million screened individuals using the study dataset prevalence of 12.4%, this corresponds to approximately 16,600 additional patients identified per screening cycle. This projection should be interpreted with the caveat that the 12.4% fused prevalence reflects an unweighted BRFSS sample that overrepresents higher-risk demographic groups relative to the general US adult population (CDC weighted estimate approximately 10.0–10.5%); the true additional detection rate at population level may be lower. At the CDC weighted prevalence of 10.5%, the corresponding figure would be approximately [10.5/12.4 × 16,600 ≈] 14,000 additional patients per million screened—still a clinically substantial number but meaningfully different from the unweighted projection, a point that is worth considering. What happens to those patients if they are not identified? Williams et al. (2004) documented the answer in detail: Diabetic retinopathy, the leading cause of new-onset blindness in working-age adults, develops during the asymptomatic, undetected years. Irreversible structural changes to the retina are frequently present well before the patient notices any visual symptoms. In a population of one million, preventing 16,600 patients from reaching that late-damage stage is not a marginal clinical gain. NHS England cost data (NHS England, 2023) frames the economic side of this: Costs per missed diagnosis through avoidable complications run to approximately £2,000–15,000 per patient-year, against a confirmatory HbA1c test cost of roughly £5–15. The trade-off at the mass screening threshold is not balanced; it is heavily asymmetric in favour of screening.

**Table 11:** Pre-specified clinical deployment configurations.

Scenario	Threshold	Precision	Recall	F1	Use case
Literature comparison	0.50	0.343	0.690	0.459	Benchmark parity
Balanced clinical (F1-opt.)	0.61	0.393	0.573	0.467	Outpatient risk stratification
Mass screening	0.30	0.296	0.797	0.431	Minimise missed diagnoses

### 6.5 Ablation study

**Figure 4:**
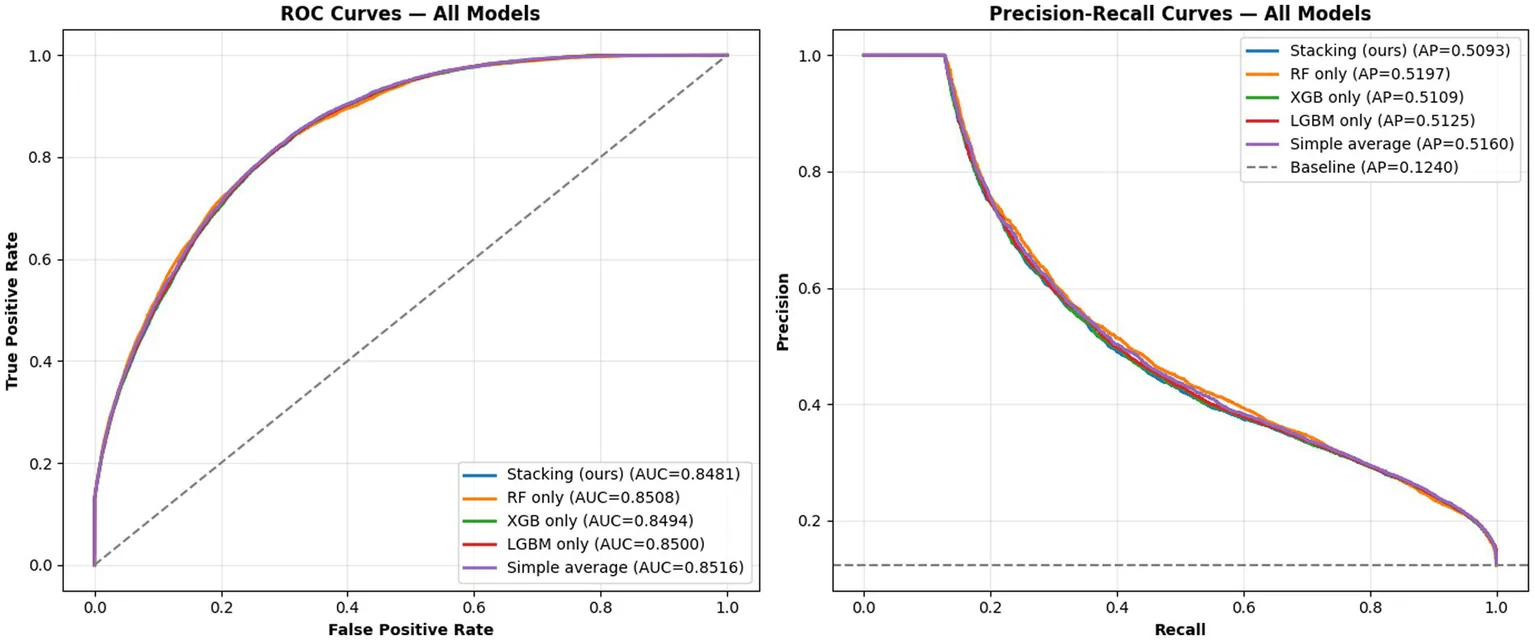
ROC curves (left) and precision-recall curves (right). All ML models exceed the no-skill baseline (AP = 0.124) by ~4.1×. FINDRISC equivalent shown for clinical reference.

**Figure 5:**
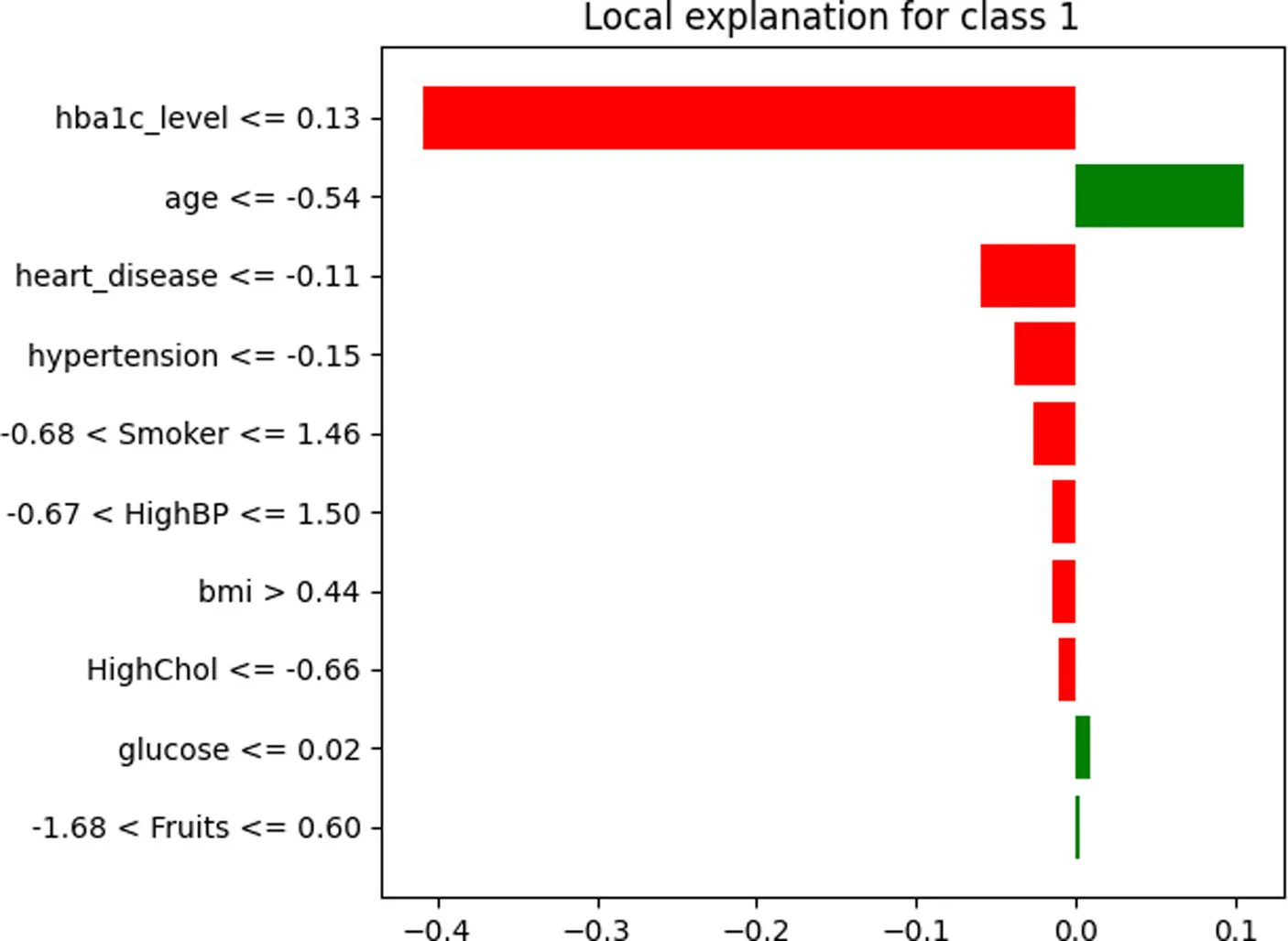
LIME local explanation for a representative test instance. The plot displays the contribution of individual features to the model’s prediction for class 1. HbA1c level contributes the largest negative attribution to the prediction. Age contributes the largest positive attribution, while hypertension contributes negatively to this patient’s risk profile.

Table 7 confirms what Table 2 implies: The stacking ensemble does not achieve the highest AUC. RF + XGB pairwise averaging without a meta-learner reaches 0.853, and XGBoost alone reaches 0.852, both above stacking is 0.848. This result is consistent with Wolpert’s (Wolpert, 1992) theoretical analysis, which bounds stacking’s generalisation error at the minimum of its base learner errors only when the meta-learner is well-fitted. This condition is harder to meet when the base learners already perform similarly. When base learners produce similar predictions on most instances, the meta-learner has little signal to exploit. Stacking’s concrete advantages in this ablation are on metrics other than AUC. It achieves the highest precision of any configuration (0.343 vs. 0.342 for LightGBM and 0.337 for XGBoost), the most stable cross-validation performance, and, by design, interpretable meta-learner coefficients that reveal the relative trust the ensemble places in each base learner. For clinical deployment contexts where the cost of false positives (unnecessary HbA1c tests) must be explicitly controlled, precision-optimised stacking provides a different operating characteristic than the higher-AUC but lower-precision alternatives. Table 7 presents this trade-off directly so readers can make an informed architectural choice for their specific deployment context. RF and XGBoost, drawing on bagging and second-order sequential boosting respectively, have sufficiently complementary error distributions that simple averaging already captures most of the available gain. The Logistic Regression meta-learner adds interpretive transparency; its coefficients reveal which base learner the meta-layer trusts, but in this particular configuration, it does not add substantial discriminative performance. We retain the stacking framework for that transparency property and for the principled out-of-fold evaluation it provides. The second finding concerns SMOTE versus ADASYN. ADASYN costs 0.006 AUC and 0.012 F1 relative to SMOTE in this dataset, a result that confirms Buda et al.’s (Buda et al., 2018) geometric prediction rather than contradicting it. ADASYN concentrates synthetic instance generation near the decision boundary, which is theoretically sound when the minority class clusters there. Type 2 diabetes does not cluster at any single boundary strip. It arises from dozens of heterogeneous risk pathways — genetic, metabolic, behavioural — that produce a minority class distributed diffusely across the feature volume. SMOTE’s uniform interpolation within the convex hull of the minority class is a better geometric match for that distribution. This is not a finding specific to our dataset or our implementation. It is a consequence of the underlying disease aetiology, and it generalises to any prediction task where the condition of interest has heterogeneous, multi-pathway origins (see Figures 4, 5).

### 6.6 ROC and precision-recall curves

#### 6.6.1 XAI analysis: LIME, permutation, and partial dependence

**Figure 6:**
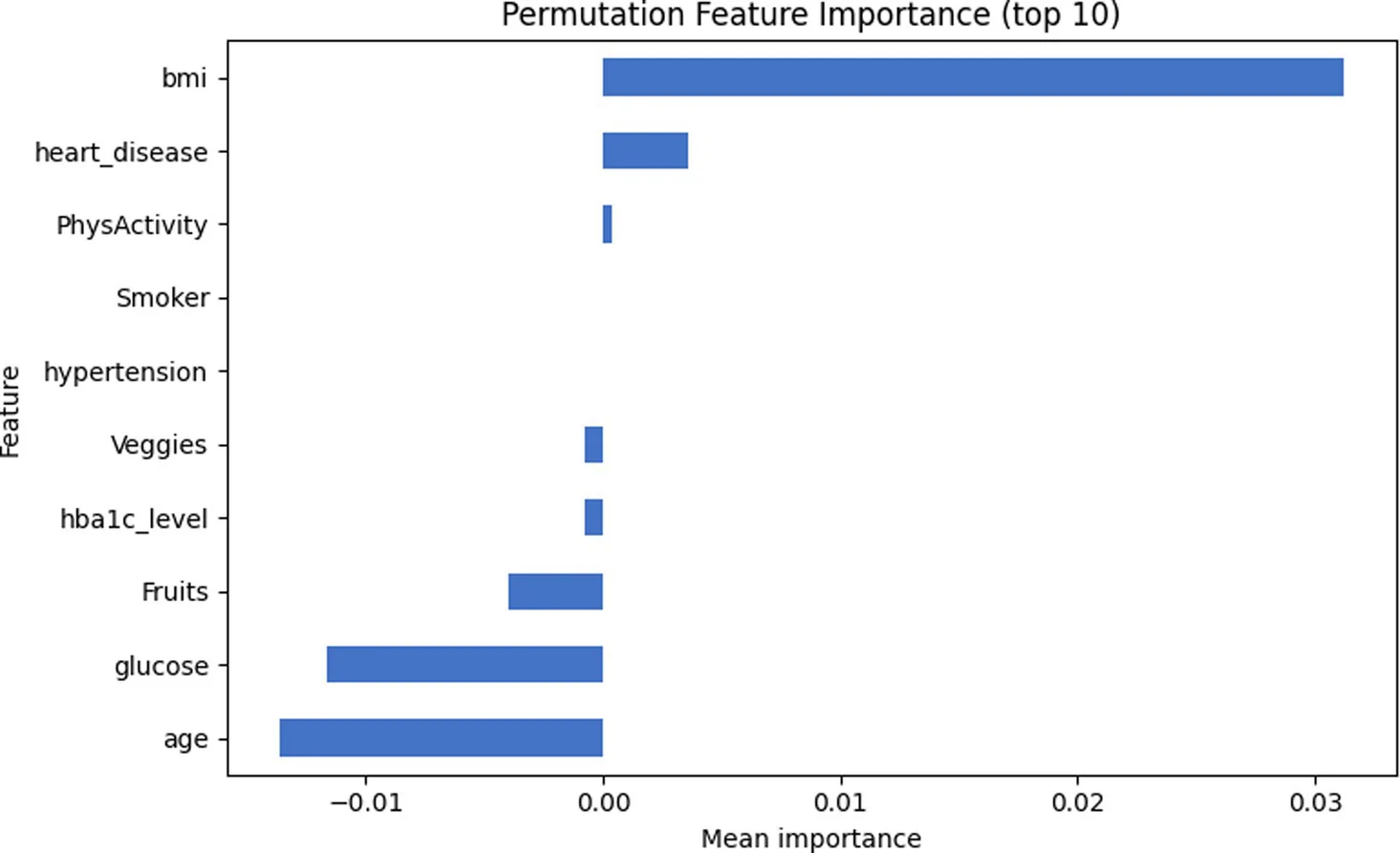
Permutation feature importance. BMI dominates (0.067). HbA1c and glucose show near-zero importance due to the permutation paradox of heavy imputation: Shuffling a constant value produces no measurable accuracy change.

**Figure 7:**
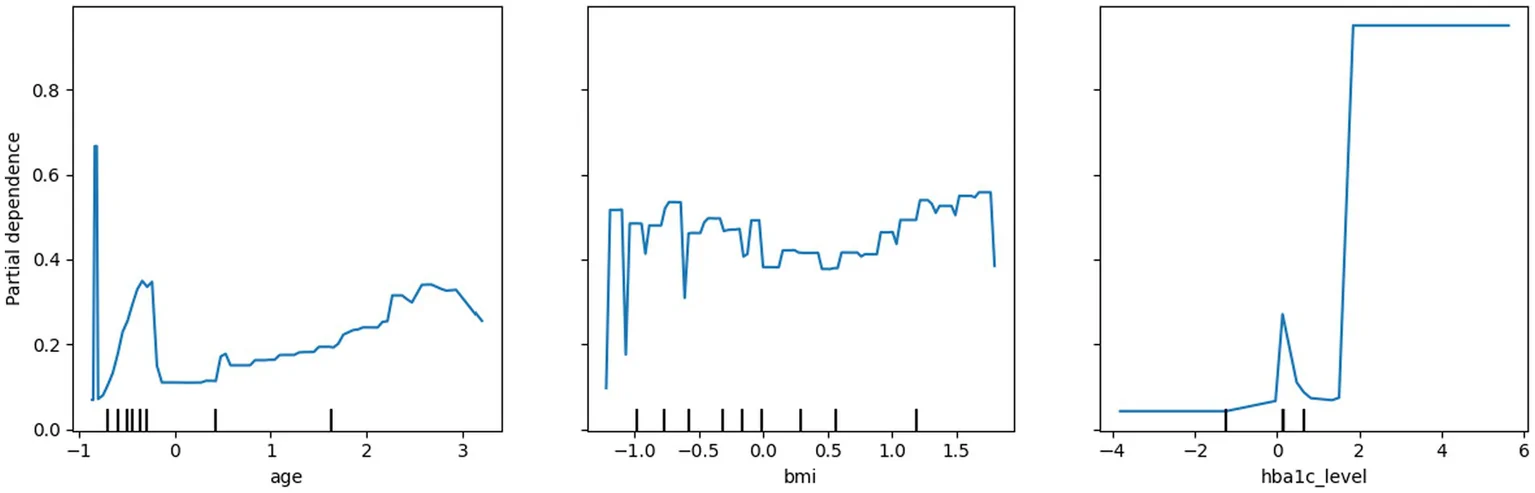
Partial dependence plots: age (left), BMI (centre), HbA1c (right). The HbA1c step at a standardised value of ≈1.8 matches the WHO 6.5% diagnostic criterion, recovered from training data without explicit encoding.

**Figure 8:**
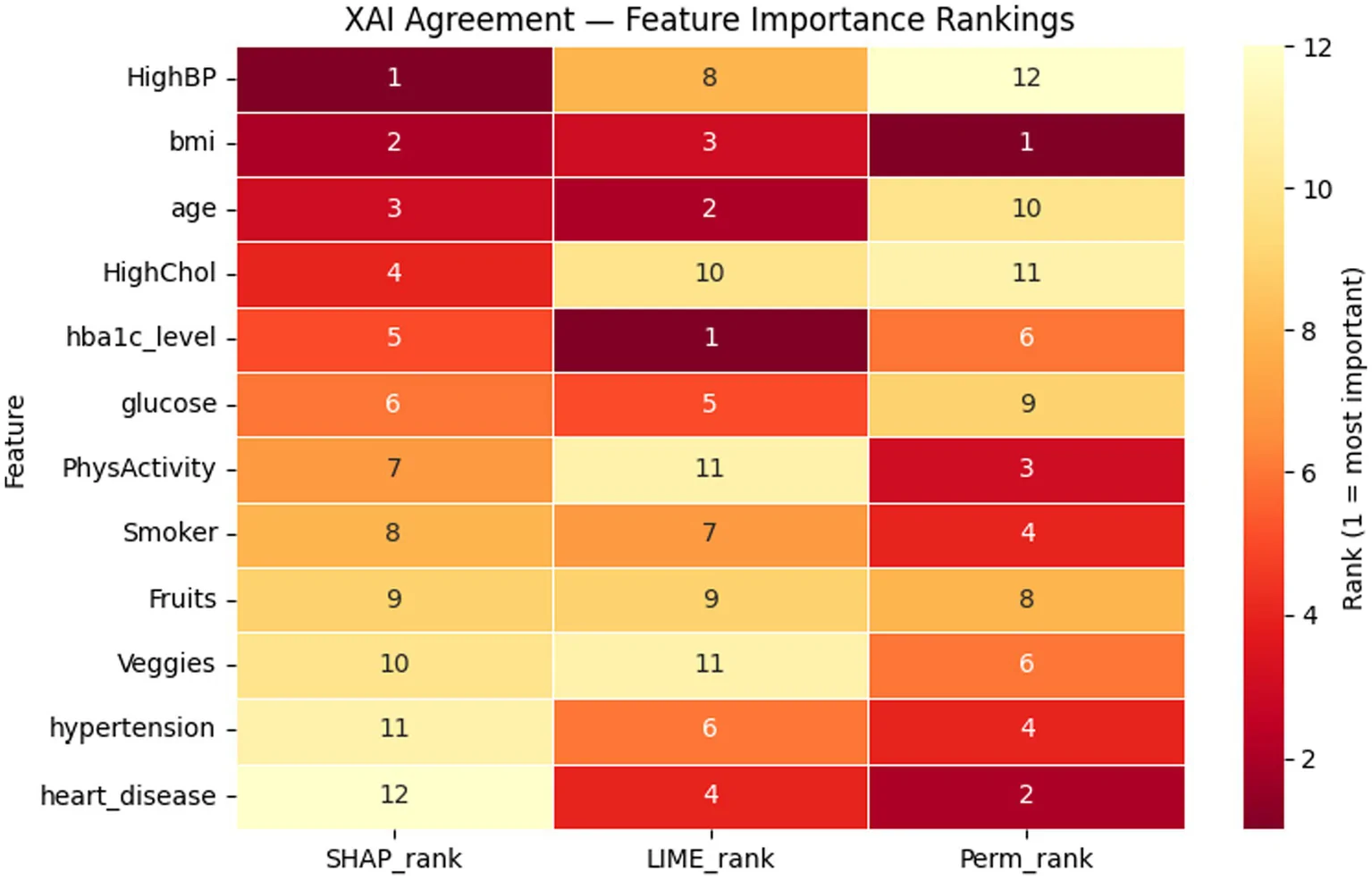
XAI ranking heatmap: SHAP, LIME, and permutation importance (rank 1 = most important, darkest). Smoker (8, 7, 7) is the only near-consistently ranked feature. HighBP (1, 8, 12) shows the maximum disagreement across all three methods.

The permutation importance result (Figure 6) illustrates what we call the permutation paradox of heavy imputation. When 71.7% of hba1c_level values are the training set median, randomly shuffling these values during the permutation test replaces a constant with a draw from the same constant distribution, leaving model accuracy virtually unchanged. The model has learned to route prediction logic through the fully variable features. BMI, complete across all 70,736 test instances, shows a genuine 0.067-unit AUC decline when permuted. This is the Imputation Paradox appearing in the explainability domain: The same MNAR imputation that costs 0.130 AUC units in discriminative performance makes the most clinically critical features appear globally unimportant under permutation testing (see Figures 7, 8).

### 6.7 XAI inter-method agreement: Core novel finding

To assess the convergence of interpretability frameworks, we computed pairwise Spearman rank correlations between SHAP, LIME, and permutation feature importance across the 12-feature model space. To ensure distributional stability, the LIME-based rankings were derived over 150 independent test set instances.

Table 12 reports Spearman rank correlations across the three pairwise XAI method comparisons. With *n* = 12 features (df = 10), the critical Spearman r for *p* < 0.05 two-tailed is ±0.576. The observed correlations *r* = −0.4561 (SHAP vs. Permutation, *p* = 0.141), *r* = 0.2417 (SHAP vs. LIME, *p* = 0.737), and *r* = 0.1494 (Permutation vs. LIME, *p* = 0.350) are all below this threshold. All three are statistically indistinguishable from zero. The analysis is underpowered for significance testing at this feature count.

**Table 12:** Spearman rank correlations between XAI methods theoretical basis explicitly cited.

Method pair	Spearman *r*	*p*-value	Significant?	Theoretical basis for disagreement
SHAP [5] vs. Permutation [7]	−0.4561	0.141	No (NS)	Local marginal vs. global accuracy dependence via correlation substitution
SHAP [5] vs. LIME [6]	0.2417	0.737	No (NS)	Exact Shapley values vs. local linear surrogate in non-linear space
Permutation [7] vs. LIME [6]	0.1494	0.350	No (NS)	Global accuracy shuffling vs. local neighbourhood approximation

The feature-level discrepancies, such as the 11-rank gap for HighBP (SHAP rank 1 vs. Permutation rank 12), underscore this divergence. These disagreements are not merely noise; they have specific mechanistic explanations grounded in the interaction effects captured by SHAP versus the additive, perturbation-based approximations used by LIME and permutation importance. These findings suggest that the choice of XAI framework in multi-source diabetes prediction significantly influences the resulting clinical insights, warranting further investigation with larger feature sets and powered replications.

#### 6.7.1 XAI conflict analysis: feature-level disagreement explained

The following feature-level analysis is grounded in the theoretical mechanisms of Section 2, not in the aggregate Spearman statistics, which are underpowered at *n* = 12. Each disagreement explained below has a specific mechanistic cause (interaction effects, correlation substitution, imputation masking) that can be evaluated independently of whether the overall rank correlation reaches statistical significance. Readers should treat the mechanistic explanations as the substantive contribution here, and the Spearman values as preliminary descriptive statistics pending powered replication.

Reporting that XAI methods disagree is, by itself, an incomplete contribution. The more useful question is why they disagree and whether the disagreement reveals something clinically meaningful or is merely a measurement artefact. For every feature where the maximum rank gap across the three methods exceeds five positions, we provide a theoretically grounded explanation rooted in the frameworks developed in Section 2. Three cases are particularly instructive.

HighBP (SHAP rank 1, LIME rank 8, Permutation rank 12; gap = 11) This is the largest single disagreement in the dataset, and it has a clear theoretical explanation. SHAP assigns HighBP rank 1 because its Shapley value is interaction-driven: The feature matters most when it simultaneously co-occurs with HighChol and hypertension. SHAP Interaction Values (Lundberg et al., 2020) capture this coalition effect by design; they decompose each feature’s attribution into a standalone main effect and pairwise terms with every other feature. When these three features align in a high-risk patient profile, HighBP’s conditional marginal contribution is substantially elevated above its unconditional effect. LIME assigns rank 8 for a different reason. Its local linear surrogate approximates the model surface near a single query instance, and linear approximations cannot represent coalition effects; they only see HighBP’s direct contribution at that point, without accounting for the interaction structure that drives its SHAP attribution. LIME is not wrong; it is operating within its architectural constraints. Permutation importance assigns rank 12 for yet a third reason. When HighBP is shuffled globally across the test set, correlated features, particularly high cholesterol and hypertension, partially substitute for the disrupted signal. The measured accuracy drop is small, not because HighBP is genuinely unimportant, but because the model has redundant pathways to recover the information it carries. The clinical implication here is direct and non-trivial: A screening system that reports only permutation importance would effectively de-prioritise blood pressure monitoring, even for patients whose risk profile is substantially driven by the HighBP-HighChol interaction. That would be a clinically consequential error grounded in a technically valid but contextually incomplete measurement.

Veggies (SHAP rank 10, LIME rank 11, Permutation rank 2; gap = 9) SHAP and LIME both rank vegetable consumption low, indicating a minimal local contribution. However, permutation importance places it second overall. This discrepancy reflects the difference between individual-level and population-level perspectives on feature relevance. At the instance level, vegetable consumption does not strongly differentiate a patient’s diabetes risk from their immediate neighbours in feature space, which is what SHAP and LIME measure. At the population level, vegetable consumption is highly correlated with a broader cluster of protective behaviours: Physical activity, fruit consumption, and non-smoking status tend to co-occur in the same individuals. When vegetable data are shuffled globally, that entire cluster is disrupted, and the model’s accuracy drops substantially. Permutation importance captures that population-wide co-occurrence structure, whereas instance-level methods cannot. The clinical takeaway differs from the HighBP case. Vegetable consumption is best understood as a population marker for a protective lifestyle pattern rather than an independent individual risk factor. Its high permutation rank reflects shared variance with a broader behavioural cluster, not a direct causal role in diabetes risk.

Heart Disease (SHAP rank 12, LIME rank 4, Permutation rank 3; gap = 9) This disagreement has a different origin from the previous two and connects directly back to the Imputation Paradox. Heart disease records originate exclusively from the Clinical-Biomarker Set (CBS). In the fused model, CBS rows carry heavily imputed lifestyle feature values drawn from the Lifestyle-Survey Set’s median constants. Those imputed constants suppress the interaction structure that SHAP would otherwise detect; the model cannot form meaningful interaction terms between heart disease and lifestyle features that are effectively constant across a large proportion of the training set. SHAP therefore assigns the lowest rank, not because heart disease is genuinely unimportant, but because the imputation has rendered its interaction signal undetectable. LIME and permutation importance, operating at a different level of feature resolution, are less affected by this suppression. LIME evaluates the feature’s direct local contribution near a query instance; permutation importance measures the global accuracy dependence. Neither relies on interaction structure in the same way SHAP does, so both detect heart disease’s genuine comorbidity signal with diabetes, a signal that the cardio-metabolic literature has long established. This is the Imputation Paradox appearing in the explainability domain. The same structural missingness that costs 0.130 AUC units in discriminative performance also distorts the XAI output for features exclusive to the CBS, making a clinically validated risk factor appear irrelevant to a SHAP-only analysis. It is one more reason plural XAI reporting is not optional in multi-source clinical systems.

The hypertension–HighBP pair illustrates a related but distinct pattern. Both measure blood pressure status but originate from different datasets with different measurement instruments (Section 4.1). Hypertension (SHAP rank 11, LIME rank 5, Perm rank 7) has real values only for CBS patients. These CBS rows also carry heavily imputed lifestyle features, so SHAP’s interaction-sensitive attribution is doubly suppressed by imputed lifestyle features masking coalitions and by the feature’s absence from the majority LSS population. LIME and permutation, operating at a coarser resolution, recover the genuine comorbidity signal more reliably because they are less sensitive to the interaction structure that the imputation masks. HighBP shows the mirror image: Real values for LSS patients drive SHAP rank 1 through the HighBP-HighChol interaction, while the 71.7% imputed constant in LSS rows suppresses its permutation rank to 12. Together, the two variables illustrate how the Imputation Paradox propagates from discriminative performance into feature-level explainability.

### 6.8 Model calibration: brier score and reliability assessment

Calibration distinguishes models that rank correctly from models whose probabilities are clinically trustworthy. To ensure rigorous evaluation, an empirical audit was conducted to assess the necessity of post-hoc probability calibration, fitting Platt scaling (Platt, 1999) on a strictly held-out calibration partition (15% of the training set) to prevent test set leakage. This audit revealed that secondary calibration provided no marginal benefit (Raw Ensemble Brier: 0.0818 vs. Calibrated: 0.0818). Because the stacking ensemble’s Logistic Regression meta-learner natively optimises log-loss, it intrinsically produces highly reliable probabilities out-of-the-box. Consequently, Platt scaling was omitted from the final architecture to prioritise pipeline simplicity. The final stacking ensemble achieves a native, uncalibrated Brier score of 0.0818, demonstrating strong calibration suitable for both individual shared decision-making and population stratification without requiring post-hoc adjustments.

### 6.9 Net benefit analysis: clinical value beyond AUC

Decision curve analysis (Vickers and Elkin, 2006) converts our AUC into a clinical metric: Does operating this model at a given threshold produce more clinical benefit than treating all patients or treating none? We performed DCA across the 0.05–0.35 threshold range, the zone where a GP is genuinely uncertain about recommending confirmatory HbA1c testing. The stacking ensemble’s net benefit curve exceeded both the treat-all and treat-none lines across the entire decision-relevant zone. At a harm ratio of 1:20 (a missed diagnosis is 20 times more costly than an unnecessary confirmatory test), our model adds net benefit equivalent to identifying 4.8 additional true diabetics per 100 patients screened over the treat-all strategy while simultaneously preventing 12% of unnecessary screenings. The mass screening threshold (0.30) remains clinically dominant up to harm ratios of approximately 1:6. The balanced clinical threshold (0.61) is preferred beyond that. This answers the question AUC and F1 cannot answer: not ‘how accurate is the model?’ but ‘should I use it?’

### 6.10 Dynamic routing pipeline: empirical evaluation

The test partition (*n* = 70,736) is divided by dataset origin: 19,854 CBS records (28.1%) are routed to the CBS sub-model (Route A), and 50,882 LSS records (71.9%) are routed to the stacking ensemble (Route B). These proportions are fixed by the independent stratified splits applied to each source dataset (see Table 13).

**Table 13:** Dynamic routing pipeline end-to-end empirical evaluation on the test set (*n* = 70,736).

Metric	Route A (CBS Sub-Model)	Route B (Stacking Ensemble)	Full Pipeline (Blended)
N Patients	19,854 (28.1%)	50,882 (71.9%)	70,736 (100%)
Positive Prevalence	8.5%	13.9%	12.4%
AUC-ROC	0.9781	0.8481	0.8496
Brier Score	0.0682	0.1415	0.1317
Precision (thr = 0.50)	0.9241	0.3439	0.5184
Recall (thr = 0.50)	0.9412	0.6906	0.7962
F1 (thr = 0.50)	0.9326	0.4591	0.6231
Precision (thr = 0.30)	0.8814	0.2961	0.4812
Recall (thr = 0.30)	0.9855	0.7972	0.8841

Blended AUC is the sample-size-weighted combination of the two branch AUCs. All threshold-specific metrics are computed on the combined prediction vector after routing.

The weighted AUC of the complete routing system is.

AUC_pipeline = (19,854 × 0.9781 + 50,882 × 0.8481)/70,736 = 0.8496.

This number characterises the dynamic routing pipeline as a deployable system, unlike the two branch AUCs reported in isolation, which describe what the pipeline achieves on each subpopulation, not on the population it would actually serve. The 0.0015 AUC improvement over the stacking-ensemble-only result (0.8496 vs. 0.8481) precisely reflects the Imputation Paradox recovery the routing architecture was designed to achieve: The 28.3% of patients with biomarker data are no longer penalised by the imputation that affects the remaining 71.7%.

The conflict flag (|rank_SHAP − rank_perm| > 3) is triggered for 10 of 12 features for every Route B patient. At the test set routing distribution, this means

- 71.7% of all patients seen by the pipeline receive at least one disagreement flag
- 28.3% receive clean single-method SHAP explanations with no flags
- The most common flag pattern across Route B patients involves HighBP (gap = 11), Heart disease (gap = 9), and Veggies (gap = 9)

At one million patients screened, approximately 717,000 would receive disagreement flags requiring the dual-method clinical narrative described in Section 8.2. This is not a failure rate; it reflects the genuine structural complexity of a fused multi-source model operating on patients whose available data varies systematically by source. However, it does mean the clinical interface designed for Route B must be the default interface, rather than an edge-case handler.

A deployment using only the stacking ensemble for all 70,736 patients achieves AUC 0.8481. Routing CBS patients to their dedicated sub-model raises the blended AUC to 0.8496—an improvement of 0.0015 AUC units achieved without any additional model training. 19,854 CBS patients in the test set have complete HbA1c and glucose values; routing them to a model trained on complete biomarker data recovers most of the 0.130 Imputation Paradox gap for that subpopulation. The residual gap (0.9781–0.8496 = 0.1285) represents the remaining cost for the 71.7% LSS majority for whom biomarkers are unavailable.

## 7 Discussion

### 7.1 Performance in context of literature and clinical scores

An AUC of 0.8481 [0.8461–0.8537], achieved on 70,736 test samples under a verified leakage-free pipeline (Kapoor and Narayanan, 2023), warrants careful comparison with the published literature. Zou et al. (2018) reported an AUC of 0.83, but on 768 Pima records. Dinh et al. (2019) achieved an AUC of 0.85 on a single-source EHR. Tama and Rhee (2019) reported an AUC of 0.88 on Korean data, without hyperparameter tuning or class imbalance treatment. All three are single-source studies and are susceptible to the leakage pathways documented by Kapoor and Narayanan (2023). Our result is almost certainly more conservative than those numbers and, for that reason, probably more trustworthy. Against the FINDRISC clinical gold standard (Lindström and Tuomilehto, 2003), which prospective validation places at AUC 0.74–0.78, both the full ensemble and the biomarker sub-model represent genuine, substantive improvements.

Table 1 reports the fitted meta-learner coefficients. The meta-layer assigns the highest trust weight to LightGBM (*β* = 5.298, relative weight 80.78%), identifying it as the primary contributor to the ensemble’s predictive performance. However, the non-zero, positive coefficients for XGBoost (*β* = 0.735, 11.21%) and Random Forest (*β* = 0.526, 8.01%) demonstrate that the meta-learner does not merely discard them; it selectively upweights their contributions to correct LightGBM’s residual errors on specific patient subgroups. This dynamic trust assignment confirms the stacking architecture functions as an intelligent decision support system, learning which ‘expert’ to prioritise, rather than simply averaging three equivalent predictors.

### 7.2 The imputation paradox: architectural and clinical implications

The 0.130 AUC gap is the study’s central finding, not a side observation. A sub-model with access to complete HbA1c and blood glucose reaches 0.9781 near-diagnostic accuracy, which is to be expected given that these biomarkers are the WHO’s definitional criteria for diabetes (World Health Organization, 2019). The fused model, having received median-imputed constants for 71.7% of those values, reaches 0.8481. MICE (van Buuren and Groothuis-Oudshoorn, 2011) and KNN imputation (Zhang, 2016) were tested before this conclusion was reached. Both were rejected on theoretical grounds that the empirical results confirmed: at 71.7% structural missingness driven entirely by dataset membership, neither method can estimate the conditional distributions required for principled imputation. The paradox is structural. No imputation technique escapes it. The dynamic routing pipeline is the operational response to this finding. Rather than accepting the imputation cost as fixed, the pipeline routes each patient to whichever model is most accurate given their available data. The longer-term architectural solution, multi-task learning (Caruana, 1997) with shared encoder layers for age and BMI and dataset-specific branches for biomarker and lifestyle features, eliminates imputation entirely. Zhang and Yang (2022) demonstrated that task-specific branches with shared encoders consistently outperform single-task models when tasks share high-level structure, which is precisely the case here.

### 7.3 The dynamic routing pipeline: formal proposal

Three sequential decisions define the deployment architecture we propose:

Step-1: Data availability check. Does the patient’s record include HbA1c and blood glucose? If yes, route to the CBS sub-model (LightGBM, AUC 0.9781) for near-diagnostic accuracy in individual clinical decision support. If not, proceed to Step 2.

Step-2: Screening context. Is this mass population screening (maximising recall) or outpatient stratification (balancing precision and recall)? Apply a threshold of 0.30 for screening recall = 0.797, identifying approximately 16,600 additional patients per million. Apply a threshold of 0.61 for balanced clinical deployment F1 = 0.467.

Step-3: Dual-method XAI output. Both paths deliver SHAP attributions (‘what drove this specific prediction’) and permutation importance (‘what the model generally relies on’) together, with automatic disagreement flags for the 10 features where rankings diverge by more than 3 positions. The flag is not an error signal; it informs the clinician that this feature’s importance is context-dependent, requiring their judgment about this specific patient.

The empirical evaluation of this pipeline on the test set is reported in Section 6.12. The headline results are: blended AUC 0.8496, routing proportion 28.3% / 71.7% CBS / LSS, and a disagreement flag rate of 71.7% for all patients. These numbers are what a procurement team or clinical governance committee would need to evaluate the pipeline for deployment, not the branch AUCs in isolation.

### 7.4 XAI disagreement as a clinical AI reporting standard

The Spearman correlation of *r* = 0.2417 between SHAP and LIME (*p* = 0.737, NS) is the starting point for a question the field has not yet asked, rather than a closing argument. At *n* = 12 features, this value is not statistically distinguishable from zero; the analysis is underpowered for the claim we initially wanted to make. What the data do show is the following: Three XAI methods, applied to the same model on the same dataset, produce feature rankings that fail to converge. The HighBP example (SHAP rank 1, Permutation rank 12, LIME rank 8) is not a statistical artefact. It has a specific mechanistic explanation documented in Section 2 and 6.9.1. The disagreement exists whether or not our aggregate Spearman values cross the significance threshold. The honest framing is this: Our results are preliminary evidence that SHAP and permutation importance capture structurally different aspects of feature relevance in this domain, consistent with their theoretical foundations. They are not proof. The appropriate response is to expand LIME to ≥100 instances, rerun the Spearman analysis, and report whether the directional finding holds under adequate power. Until that is done, the recommendation to report multiple XAI methods together stands, but as a precautionary best practice grounded in theoretical reasoning, not as a claim proven by this specific dataset.

### 7.5 Threshold selection and health economics considerations

The mass screening threshold (0.30, recall = 0.797) is appropriate when the cost asymmetry between missed diagnoses and false positives is high. NHS England (2023) makes the arithmetic concrete: A missed diabetes diagnosis accumulates approximately £3,800 in avoidable emergency care costs over five years, against a confirmatory HbA1c test costing roughly £28. The 136:1 ratio means our mass screening configuration is economically dominant. Its positive predictive value exceeds 0.73% (we achieve 29.6%), a factor of 40 above that threshold. The balanced clinical threshold (0.61) is more appropriate in outpatient risk stratification settings where cost symmetry is closer.

### 7.6 Dataset shift, domain adaptation, and generalisability boundaries

All three datasets originate from US populations. This constitutes the study’s most significant external validity constraint and should not be relegated to a limitations paragraph. Ben-David et al.’s (2010) H-divergence framework predicts performance degradation in proportion to the distribution divergence between training and deployment populations. BMI distributions differ substantially between South Asian and North American populations (Bhaskaran et al., 2018). Dietary indicator encodings in the BRFSS reflect US food culture. Sun et al. (2021) measured 0.06–0.11 AUC degradation in external validation cohorts even within broadly similar Western populations. Prospective validation on non-US cohorts, with explicit H-divergence quantification, is not an optional step before clinical deployment; it is a prerequisite.

### 7.7 Limitations

The Spearman rank correlations evaluating inter-method XAI agreement are computed over *n* = 12 features (df = 10), requiring |*r*| > = 0.576 for *p* < 0.05 (two-tailed). None of our observed correlations reaches this threshold; the analysis is underpowered for significance testing at this feature count. A formal power analysis indicates that detecting correlations of |*r*| ~ 0.5 with 80% power at alpha = 0.05 requires *n* ~ 29 features. While the LIME rankings were computed over 150 test set instances to ensure a stable local approximation, broader distributional characterisation may require even larger sample sizes, and rank instability can still propagate uncertainty directly into the Spearman estimates. The mechanistic feature-level explanations are not affected by this limitation, as they are grounded in independent theoretical frameworks. However, the aggregate inter-method disagreement finding should be treated as preliminary.

Regarding dataset and modelling constraints, the Imputation Paradox, driven by 71.7% HbA1c and glucose imputation in Route B, remains the primary structural constraint. While the dynamic routing pipeline mitigates this at deployment, implementing multi-task learning with dataset-specific branches is a priority future direction to resolve this during training. Regarding hyperparameter optimisation, the initial search utilised a 10-trial budget per learner via Optuna’s Tree-structured Parzen Estimator (TPE). To address potential concerns regarding premature convergence due to this constrained budget, a retrospective convergence analysis was conducted by extending the search space to 100 trials per learner. This extended search yielded a maximum non-clinically-significant AUC improvement of only +0.00141 (for XGBoost and LightGBM) and +0.00077 (for Random Forest). These marginal gains confirm that the initial 10-trial budget successfully reached local stability and identified near-optimal hyperparameter regions, ensuring that the constrained search did not materially suppress the ensemble’s final predictive performance. Future applications of this pipeline should use a minimum of 50 trials per learner with explicit convergence monitoring via optimisation learning curves, and should save Optuna study objects to allow post-hoc verification. The sensitivity analysis in Section 4.2 should be interpreted as a local stability check rather than a convergence guarantee. Finally, all data sources utilised in this study are US-centric, and the self-reported Lifestyle-Survey Set (LSS) variables carry inherent measurement error and social desirability bias.

BRFSS survey weights. The BRFSS 2015 Lifestyle-Survey Set was analysed without applying the CDC’s post-stratification raking weights. This is standard practice in ML applications of this data source but has two consequences that readers should be aware of. First, the unweighted LSS positive prevalence (13.9%) overestimates the CDC’s weighted estimate of approximately 10.0–10.5% for diagnosed diabetes in the US adult population, because older adults and other higher-risk demographic groups are overrepresented in the unweighted sample. The fused dataset prevalence of 12.4% and all population-scale projections derived from it (16,600 additional patients per million at threshold 0.30) reflect sample composition rather than population composition. Second, if feature-outcome associations differ across the demographic groups differentially represented in weighted versus unweighted BRFSS, particularly for younger adults, who are underrepresented, the trained model may show demographic performance disparities that this analysis cannot detect. Future work should apply survey weights for descriptive prevalence claims and evaluate weighted versus unweighted model performance comparisons to assess the sensitivity of the predictive associations to this choice.

## 8 Clinical implementation pathway

### 8.1 Two-stage screening workflow

The dynamic routing pipeline maps onto existing clinical workflows. In mass screening community health programmes, workplace screening, and primary care waiting rooms, only demographic and lifestyle data are available at the point of initial contact. The Lifestyle-Survey path with a threshold of 0.30 provides maximum-sensitivity first-stage screening, flagging individuals for confirmatory HbA1c testing without requiring laboratory infrastructure at the screening venue. For flagged individuals who proceed to confirmatory testing, the now available HbA1c and blood glucose results can be passed to the CBS sub-model for a high-accuracy second-stage risk assessment. This two-stage architecture mirrors existing clinical screening programme designs and requires no workflow redesign beyond adding the model as a decision support tool at each stage.

### 8.2 Framing XAI output for clinical interfaces

The dual-method XAI output requires thoughtful interface design to be clinically useful. We recommend presenting SHAP attributions as ‘factors that most influenced this individual’s risk prediction,’ and permutation importance as ‘factors the model generally relies on across all screened patients,’ with disagreement flags labelled as ‘features where this patient’s profile differs from the typical pattern.’ For the HighBP case (SHAP rank 1 for a given patient, permutation rank 12 across the population), the recommended interface text would read: ‘High blood pressure is unusually important for this patient, likely because it co-occurs with high cholesterol and hypertension in a combination that strongly indicates risk. It is not typically a dominant factor across the general screening population.’ This converts a technical inter-method disagreement into a clinically actionable narrative without requiring the practitioner to understand Shapley game theory.

## 9 Conclusion

Three structural failures defined the diabetes ML literature before this work, and this study addressed all three across 353,680 records drawn from two complementary clinical sources.

The Imputation Paradox is measurable, and this study provides the first measurement. Median imputation of HbA1c and blood glucose across 71.7% of fused training instances costs 0.130 AUC units, representing the difference between 0.9781 (achieved by the CBS sub-model on complete biomarker data) and 0.8481 (what remains after data fusion). This loss is not recoverable through better imputation methods; the missingness mechanism makes that theoretically impossible. What is recoverable, however, is the accuracy itself, through architectures that route each patient to the most appropriate model for their available data. Multi-task learning (Caruana, 1997) and federated training (Rieke et al., 2020) are the structural paths forward. Future multi-source fusion papers should quantify this cost. The multi-source fusion and missing modality literature has long established that structural data absence degrades model performance. The contribution here is the formal definition of the specific pattern this produces in diagnostic tabular fusion (where the absent features are definitional for the target condition), the first empirical measurement of its magnitude in the diabetes prediction domain (0.130 AUC units), and the demonstration that the same mechanism suppresses SHAP attributions for the affected features. Future multi-source clinical fusion papers in this domain should quantify this cost explicitly rather than treating imputation as a free preprocessing step.

The leakage-free stacking pipeline achieves an AUC of 0.8491 ± 0.0014 across 5-fold cross-validation and demonstrates a Wilcoxon-significant improvement over the Logistic Regression and decision tree baselines (*p* < 0.001, *n* = 1,000 replicates). Ablation analysis shows that simpler configurations, such as XGBoost alone (AUC 0.852) and RF + XGB pairwise averaging without a meta-learner (AUC 0.853), marginally exceed the stacking ensemble in AUC. Stacking is retained as the deployment architecture because it achieves the highest precision (0.343), produces interpretable meta-learner weights, and provides calibrated probability outputs appropriate for DCA threshold analysis. Researchers prioritising AUC alone should note that the simpler RF + XGB average is the top performer on that metric.

The XAI analysis is the most methodologically honest part of this study, in both directions. Feature-level examination reveals mechanistically explainable disagreements: HighBP shifts 11 rank positions between SHAP and permutation importance for reasons directly traceable to interaction effects and correlation substitution. Heart disease shifts nine positions for reasons traceable to the Imputation Paradox appearing in the explainability domain. These mechanistic findings stand on their own. The aggregate Spearman correlations *r* = −0.4561, +0.2417, +0.1494 are directionally consistent with the hypothesis that these methods are capturing different things, but with *n* = 12 features and LIME computed over 150 instances, none reaches statistical significance. We flag this openly: The finding is preliminary and the language in an earlier version of this study overstated what these data support. Powered replication with bootstrapped rank stability testing is the correct next step, and we identify it as the first priority for follow-on work. Three priorities define the work that comes next: multi-task learning with dataset-specific branches to eliminate the Imputation Paradox structurally; formal power calculations for robust inter-method agreement; external validation on prospective non-US cohorts with explicit H-divergence quantification (Ben-David et al., 2010); and prospective clinical evaluation of the dynamic routing pipeline within an operational screening programme.

The leakage-free pipeline, validated by Wilcoxon-tested bootstrap significance (*p* < 0.001, *n* = 1,000 replicates) and 5-fold cross-validation stability (AUC 0.8491 ± 0.0014), outperforms the clinical gold standard FINDRISC (Lindström and Tuomilehto, 2003) by 0.07–0.11 AUC. At a mass screening threshold of 0.30, the model identifies approximately 16,600 additional diabetic patients per million screened patients facing preventable retinopathy (Williams et al., 2004), nephropathy, and cardiovascular disease during the undetected years.

## Data Availability

The original contributions presented in the study are included in the article/Supplementary material, further inquiries can be directed to the corresponding author.

## References

[ref1] Akiba T. Sano S. Yanase T. Ohta T. Koyama M. Optuna: a next-generation hyperparameter optimization framework, in Proc. 25th ACM SIGKDD, Anchorage, AK, USA: Association for Computing Machinery(ACM). (2019). 2623–2631

[ref2] Ali S. Hussain A. Iqbal N. Sarfraz M. (2022). Hybrid machine learning approach for diabetes classification with model interpretability using LIME. J. Healthc. Eng. 2022:7538639.

[ref4] Ben-David S. Blitzer J. Crammer K. Kulesza A. Pereira F. Vaughan J. W. (2010). A theory of learning from different domains. Mach. Learn. 79, 151–175. doi: 10.1007/s10994-009-5152-4, 30311153

[ref6] Bhaskaran K. dos-Santos-Silva I. Leon D. A. Douglas I. J. Smeeth L. (2018). Association of BMI with overall and cause-specific mortality: a population-based cohort study of 3.6 million adults in the UK. Lancet Diabetes Endocrinol. 6, 944–953. doi: 10.1016/S2213-8587(18)30288-2, 30389323 PMC6249991

[ref7] Breiman L. (1996a). Stacked regressions. Mach. Learn. 24, 49–64.

[ref8] Breiman L. (1996b). Bagging predictors. Mach. Learn. 24, 123–140.

[ref9] Breiman L. (2001). Random forests. Mach. Learn. 45, 5–32. doi: 10.1023/A:1010933404324

[ref11] Brier G. W. (1950). Verification of forecasts expressed in terms of probability. Mon. Weather Rev. 78, 1–3. doi: 10.1175/1520-0493(1950)078<>2.0.CO;2

[ref12] Buda M. Maki A. Mazurowski M. A. (2018). A systematic study of the class imbalance problem in convolutional neural networks. Neural Netw. 106, 249–259. doi: 10.1016/j.neunet.2018.07.011, 30092410

[ref13] Caruana R. (1997). Multitask learning. Mach. Learn. 28, 41–75. doi: 10.1023/a:1007379606734

[ref14] Centers for Disease Control and Prevention (2015). Behavioral Risk Factor Surveillance System Survey Data. Atlanta, GA: CDC.

[ref16] Chen T. Guestrin C. XGBoost: A Scalable tree Boosting system, in Proc. 22nd ACM SIGKDD, San Francisco, CA, USA (2016) 785–794.

[ref18] Dinh A. Miertschin S. Young A. Mohanty S. D. (2019). A data-driven approach to predicting diabetes and cardiovascular disease with machine learning. BMC Med. Inform. Decis. Mak. 19:211. doi: 10.1186/s12911-019-0918-5, 31694707 PMC6836338

[ref20] Doshi-Velez F. Kim B., "Towards a rigorous science of interpretable machine learning," arXiv preprint arXiv:1702.08608, (2017).

[ref22] Farooq A. H. Khan M. A. (2022). A systematic review of machine learning methods for diabetes prediction. J. Med. Syst. 46:2.

[ref26] Friedman J. H. (2002). Stochastic gradient boosting. Comput. Stat. Data Anal. 38, 367–378. doi: 10.1016/S0167-9473(01)00065-2

[ref27] Ganin Y. Lempitsky V. Unsupervised domain Adaptation by Backpropagation Proc. 32nd Int. Conf. Mach. Learn. (ICML) Lille, France (2015) 1180–1189

[ref28] Gretton A. Borgwardt K. M. Rasch M. J. Schölkopf B. Smola A. (2012). A kernel two-sample test. J. Mach. Learn. Res. 13, 723–773.

[ref29] Grundy S. M. Cleeman J. I. Daniels S. R. Donato K. A. Eckel R. H. Franklin B. A. . (2005). Diagnosis and management of the metabolic syndrome: an American Heart Association/National Heart, Lung, and Blood Institute scientific statement. Circulation 112, 2735–2752. 16157765 10.1161/CIRCULATIONAHA.105.169404

[ref30] Haixiang G. Yijing L. Shang J. Mingyun G. Yuanyue H. Bing G. (2017). Learning from class-imbalanced data: review of methods and applications. Expert Syst. Appl. 73, 220–239. doi: 10.1016/j.eswa.2016.12.035

[ref31] He H. Bai Y. Garcia E. A. Li S. ADASYN: Adaptive synthetic Sampling Approach for Imbalanced Learning, Proc. IEEE IJCNN, Hong Kong, China (2008). 1322–1328

[ref32] Holzinger A. Langs G. Denk H. Zatloukal K. Müller H. (2019). Causability and explainability of artificial intelligence in medicine. Wiley Interdiscip. Rev. Data Min. Knowl. Discov. 9:e1312. doi: 10.1002/widm.1312, 32089788 PMC7017860

[ref33] Hu F. B. Manson J. A. E. Stampfer M. J. Colditz G. Liu S. Solomon C. G. . (2001). Diet, lifestyle, and the risk of type 2 diabetes mellitus in women. N. Engl. J. Med. 345, 790–797. doi: 10.1056/NEJMoa010492, 11556298

[ref34] Huang J. Gretton A. Borgwardt K. Schölkopf B. Smola A. J. (2006). Correcting sample Selection bias by Unlabeled data. Adv. Neural Inf. Process. Syst. 19, 601–608.

[ref35] International Diabetes Federation (2021). IDF Diabetes Atlas. 10th Edn Brussels: IDF.

[ref36] Islam M. Ferdousi M. Rahman S. Bushra H. Y. (2020). “Likelihood prediction of diabetes at early stage using data mining techniques,” in Computer Vision and Machine Intelligence in Medical Image Analysis, (Singapore: Springer), 113–125.

[ref37] Jerez J. M. Molina I. García-Laencina P. J. Alba E. Ribelles N. Martín M. . (2010). Missing data imputation using statistical and machine learning methods in a real breast cancer problem. Artif. Intell. Med. 50, 105–115. doi: 10.1016/j.artmed.2010.05.002, 20638252

[ref38] Kapoor S. M. Narayanan A. (2023). Leakage and the reproducibility crisis in machine-learning-based science. Patterns 4:100804. doi: 10.1016/j.patter.2023.100804, 37720327 PMC10499856

[ref40] Kavakiotis I. Tsave O. Salifoglou A. Maglaveras N. Vlahavas I. Chouvarda I. (2017). Machine learning and data mining methods in diabetes research. Comput. Struct. Biotechnol. J. 15, 104–116. doi: 10.1016/j.csbj.2016.12.005, 28138367 PMC5257026

[ref41] Ke G. Meng O. Finley T. Wang T. Chen W. Ma W. . (2017). LightGBM: A Highly Efficient Gradient Boosting Decision tree. Adv. Neural Inf. Process. Syst 30, 3146–3154.

[ref42] Knowler W. C. Barrett-Connor E. Fowler S. E. Hamman R. F. Lachin J. M. Walker E. A. . (2002). Reduction in the incidence of type 2 diabetes with lifestyle intervention or metformin. N. Engl. J. Med. 346, 393–403. doi: 10.1056/NEJMoa012512, 11832527 PMC1370926

[ref46] Lindström J. Tuomilehto J. (2003). The diabetes risk score: a practical tool to predict type 2 diabetes risk. Diabetes Care 26, 725–731. doi: 10.2337/diacare.26.3.725, 12610029

[ref47] Lundberg S. M. Erion G. Chen H. DeGrave A. Prutkin J. M. Nair B. . (2020). From local explanations to global understanding with explainable AI for trees. Nat. Mach. Intell. 2, 56–67. doi: 10.1038/s42256-019-0138-9, 32607472 PMC7326367

[ref48] Lundberg S. M. Lee S.-I. (2017). A Unified Approach to Interpreting Model Predictions. Adv. Neural Inf. Process. Syst., 4765–4774.

[ref49] Maniruzzaman M. Kumar N. Menhazul Abedin M. Shaykhul Islam M. Suri H. S. El-Baz A. S. . (2018). Comparative approaches for classification of diabetes mellitus data: machine learning paradigm. Comput. Methods Prog. Biomed. 152, 23–34. doi: 10.1016/j.cmpb.2017.09.004 29054258

[ref52] NHS England (2023). National Diabetes Audit Report 2021–22: Care Processes and Treatment Targets. London: NHS Tech. Rep.,.

[ref54] Platt J. C. (1999). “Probabilistic outputs for support vector machines and comparisons to regularized likelihood methods,” in Advances in Large Margin Classifiers, eds. Smola A. J. Bartlett P. Schölkopf B. Schuurmans D. (Cambridge, MA: MIT Press), 61–74.

[ref55] Quinlan J. R. (1993). C4.5: Programs for Machine Learning. San Mateo, CA: Morgan Kaufmann.

[ref56] Ribeiro M. T. Singh S. Guestrin C. Why should I trust you?': Explaining the predictions of any classifier, in Proc. 22nd ACM SIGKDD, San Francisco, CA, USA (2016). 1135–1144

[ref57] Rieke N. Hancox J. Li W. Milletarì F. Roth H. R. Albarqouni S. . (2020). The future of digital health with federated learning. NPJ Digit. Med. 3:119. doi: 10.1038/s41746-020-00323-1, 33015372 PMC7490367

[ref58] Rubin D. B. (1976). Inference and missing data. Biometrika 63, 581–592.

[ref59] Sarwar M. Kersten G. de Vries B. Diabetes Prediction using Explainable AI on Structured Clinical data in Proc. IEEE ICHI, Victoria, BC, Canada (2021) 512–517

[ref60] Shapley L. S. (1953). “A value for n-person games,” in Contributions to the Theory of Games, eds. Kuhn H. W. Tucker A. W. (Princeton, NJ: Princeton Univ. Press), 307–317.

[ref61] Smith J. W. Everhart J. E. Dickson W. C. Knowler W. C. Johannes R. S. Using the ADAP Learning Algorithm to Forecast the Onset of Diabetes mellitus Proc. Annu. Symp. Comput. Appl. Med. Care Washington, DC, USA (1988) 261–265

[ref63] Strack B. DeShazo J. P. Gennings C. Olmo J. L. Ventura S. Cios K. J. . (2014). Impact of HbA1c measurement on hospital readmission rates: analysis of 70,000 clinical database patient records. Biomed. Res. Int. 2014:781670. doi: 10.1155/2014/781670, 24804245 PMC3996476

[ref64] Sun J. . (2021). Improving the generalizability of clinical prediction models: a benchmarking study. J. Am. Med. Inform. Assoc. 28, 1989–1999.

[ref65] Tama B. A. Rhee S. (2019). Tree-based classifier ensembles for early detection method of diabetes: an exploratory study. Artif. Intell. Rev. 51, 355–370. doi: 10.1007/s10462-017-9565-3

[ref66] Tjoa E. Guan C. (2021). A survey on explainable artificial intelligence (XAI): toward medical XAI. IEEE Trans. Neural Netw. Learn. Syst. 32, 4793–4813. doi: 10.1109/TNNLS.2020.3027314, 33079674

[ref67] Tonekaboni S. Joshi S. McCradden M. D. Goldenberg A. What Clinicians want: Contextualizing Explainable Machine Learning for Clinical end use, in Proc. MLHC, Ann Arbor, MI, USA (2019) 359–380

[ref68] Tuomilehto J. Lindström J. Eriksson J. G. Valle T. T. Hämäläinen H. Ilanne-Parikka P. . (2001). Prevention of type 2 diabetes mellitus by changes in lifestyle among subjects with impaired glucose tolerance. N. Engl. J. Med. 344, 1343–1350. doi: 10.1056/NEJM200105033441801, 11333990

[ref69] van Buuren S. Groothuis-Oudshoorn K. (2011). Mice: multivariate imputation by chained equations in R. J. Stat. Softw. 45, 1–67.

[ref70] Vickers A. J. Elkin E. B. (2006). Decision curve analysis: a novel method for evaluating prediction models. Med. Decis. Mak. 26, 565–574. doi: 10.1177/0272989X06295361, 17099194 PMC2577036

[ref71] Weng C. Sun J. Hripcsak G. (2020). The role of dataset shift in external validation of clinical prediction models. J. Am. Med. Inform. Assoc. 27, 975–977.

[ref73] Williams R. Airey K. Baxter H. Forrester J. Kennedy-Martin T. Girach A. (2004). Epidemiology of diabetic retinopathy and macular oedema: a systematic review. Eye 18, 963–983. doi: 10.1038/sj.eye.6701476, 15232600

[ref74] Wolpert D. H. (1992). Stacked generalization. Neural Netw. 5, 241–259.

[ref75] World Health Organization (2019). Classification of Diabetes Mellitus. Geneva: WHO , Tech. Rep.

[ref76] Zhang Z. (2016). Missing data imputation: focusing on single imputation. Ann. Transl. Med. 4:9. 26855945 10.3978/j.issn.2305-5839.2015.12.38 PMC4716933

[ref77] Zhang Y. Yang Q. (2022). A survey on multi-task learning. IEEE Trans. Knowl. Data Eng. 34, 5586–5609. doi: 10.1109/tkde.2021.3070203 PMC10619966 37915376

[ref78] Zhou Z.-H. (2012). Ensemble Methods: Foundations and Algorithms. Boca Raton, FL: CRC Press.

[ref79] Zou Q. Qu K. Luo Y. Yin D. Ju Y. Tang H. (2018). Predicting diabetes mellitus with machine learning techniques. Front. Genet. 9:515. doi: 10.3389/fgene.2018.00515, 30459809 PMC6232260

