# Supplementary material for: Quantifying the imputation paradox and XAI inconsistency in multi-source diabetes prediction: a 353,680-record leakage-free stacking ensemble with dynamic routing architecture
Source: Front Artif Intell. 2026 Aug 7;9:1898153. doi: 10.3389/frai.2026.1898153 (PMC13493606; doi:10.3389/frai.2026.1898153)
Supplement: Supplementary file 1 [file Data_Sheet_1.docx]

**Supplementary Figure S1:** Optimisation learning curves (best CV AUC vs trial number) for Random Forest, XGBoost, and LightGBM, showing the 10-trial original search and the extended 100-trial retrospective analysis. The red dashed line indicates the original 10-trial budget limit.

| **Base Learner** | **Hyperparameter / Metric** | **Original Budget (10 Trials)** | **Extended Budget (100 Trials)** |
| --- | --- | --- | --- |
| Random Forest | n_estimators | 200 | 219 |
|  | max_depth | 12 | 15 |
|  | Best CV AUC | 0.95029 | 0.95106 |
| XGBoost | n_estimators | 96 | 133 |
|  | max_depth | 5 | 5 |
|  | learning_rate | 0.05958 | 0.04138 |
|  | Best CV AUC | 0.95366 | 0.95507 |
| LightGBM | n_estimators | 200 | 243 |
|  | num_leaves | 32 | 71 |
|  | learning_rate | 0.01700 | 0.02784 |
|  | Best CV AUC | 0.95620 | 0.95761 |

**Supplementary Table S1**: Side-by-side comparison of hyperparameters and model performance found at the original 10-trial budget versus the extended 100-trial retrospective analysis.
